# Low‐Density Lipoprotein Receptor‐Targeted Biomimetic Nanoplatform Suppresses Glioblastoma Stemness and Remodels Antitumor Immunity via Dual Metabolic Disruption

**DOI:** 10.1002/advs.78189

**Published:** 2026-10-11

**Authors:** Yining Jiang, Wenzhuo Yang, Wenting Cheng, Jing Zuo, Lin Pan, Sheng Zhong, Ren Geng, Yao Zhao, Zijian Chen, Canhua Huang, Yang Bai, Liyan Zhao, Yunqian Li

**Affiliations:** ^1^ Department of Neurosurgery, Translational Medicine Research Institute First Hospital of Jilin University Changchun People's Republic of China; ^2^ Department of Biotherapy, Institute of Oxidative Stress Medicine, Cancer Center and State Key Laboratory of Biotherapy, West China Hospital and West China School of Basic Medical Sciences and Forensic Medicine Sichuan University Chengdu People's Republic of China; ^3^ Department of Neurosurgery, State Key Laboratory of Oncology in South China, Collaborative Innovation Center for Cancer Medicine Sun Yat‐sen University Cancer Center Guangzhou 510060 People's Republic of China; ^4^ Department of Blood Transfusion Second Hospital of Jilin University Changchun People's Republic of China; ^5^ Department of Neurology, Neuroscience Research Center The First Hospital of Jilin University Changchun People's Republic of China; ^6^ Frontiers Medical Center Tianfu Jincheng Laboratory Chengdu People's Republic of China; ^7^ Department of Surgical Oncology The First Affiliated Hospital of Kunming Medical University Kunming People's Republic of China

**Keywords:** blood–brain barrier, cholesterol homeostasis, glioblastoma, glioblastoma stemness, metabolic intervention, tumor immune remodeling

## Abstract

Glioblastoma (GBM) remains one of the most lethal malignancies because durable tumor control is constrained by limited brain‐tumor drug exposure, metabolic plasticity, stem‐like tumor states, and an immunosuppressive microenvironment. We developed apolipoprotein B fragment‐decorated nanoparticles co‐loaded with regorafenib and erythrodiol (ApoBf‐R/E@NPs), a low‐density lipoprotein receptor‐oriented biomimetic nanoplatform that couples brain‐tumor delivery with coordinated dual‐drug exposure. Fixed‐ratio combination‐index analysis demonstrated synergy between the two agents. Regorafenib disrupts autophagy‐associated nutrient salvage and restricts angiogenic support, whereas erythrodiol stabilizes ATP‐binding cassette transporter A1 and depletes cellular cholesterol. These complementary perturbations converge on ATP depletion, mitochondrial dysfunction, and suppression of phosphoinositide 3‐kinase/protein kinase B and mitogen‐activated protein kinase signaling. Across established and patient‐derived GBM models, treatment broadly impaired tumor‐cell survival while suppressing stemness‐associated programs and reducing viability and tumorsphere persistence in patient‐derived glioma stem‐like cell cultures. Treatment also promoted an immunogenic cell death‐associated tumor‐cell state and antitumor immune remodeling. In immunocompetent GBM models, ApoBf‐R/E@NPs showed preferential intracranial tumor‐associated delivery, suppressed orthotopic and untreated distal tumor growth, and delayed postoperative regrowth, accompanied by systemic immune remodeling and expansion of memory‐associated T‐cell populations. These findings establish a blood‐brain barrier‐oriented nanodelivery strategy that couples complementary metabolic vulnerabilities to broad GBM control, stem‐like compartment suppression, and antitumor immunity.

## Graphical abstract

A low‐density lipoprotein receptor‐oriented biomimetic nanoplatform couples brain‐tumor delivery with a mechanistically matched regorafenib‐erythrodiol combination. Concurrent disruption of autophagic nutrient salvage and cholesterol homeostasis collapses bioenergetic and signaling support, suppresses glioblastoma stemness, and promotes immunogenic tumor‐cell stress and antitumor immune remodeling, thereby controlling intracranial disease and delaying postoperative regrowth across complementary preclinical models.

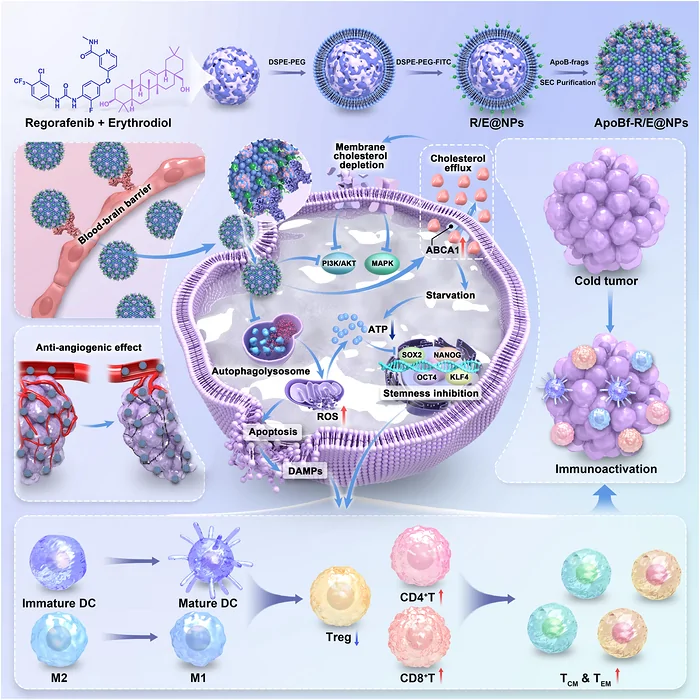

## 1 Introduction

Glioblastoma (GBM) remains the most intractable primary malignancy of the central nervous system and is characterized by diffuse tissue infiltration, metabolic plasticity, and pronounced therapeutic resistance [1]. Despite maximal surgical resection followed by intensive radiochemotherapy, tumor recurrence remains virtually inevitable [2]. Durable tumor control is further constrained by the blood‐brain barrier (BBB), which limits effective exposure of systemically administered therapeutics within the brain tumor [3]. Within the heterogeneous malignant population, glioma stem‐like cells (GSCs) represent an important contributor to treatment failure because stem‐like tumor states can support tumor repopulation after therapy and participate in the establishment of an immunosuppressive tumor microenvironment (TME) [4, 5]. By limiting effector T‐cell infiltration and promoting immunosuppressive cellular networks, stem‐like glioma cells contribute directly to immune exclusion [6]. Accordingly, effective therapy must suppress the broader GBM population while retaining activity against the stem‐like compartment that contributes to recurrence and immune escape [7]. This places brain‐tumor drug delivery and control of malignant cell states within the same therapeutic problem.

Recent nanomedicine strategies for GBM have improved intracranial drug access through receptor‐mediated transport, biomimetic carriers, barrier‐responsive surface engineering, and physical modulation of the BBB [8], with several platforms further integrating chemotherapy, photodynamic/sonodynamic therapy, or immunotherapy [9]. These advances establish that intracranial drug access can be engineered, but the more consequential challenge is whether improved brain‐tumor delivery can be translated into effective engagement of the biological dependencies that sustain both the broader GBM population and its stem‐like states [10]. We therefore sought to couple BBB‐directed delivery with a mechanistically matched metabolic intervention, so that enhanced tumor access would place complementary metabolic pressure within the malignant compartment rather than merely increase intracranial drug exposure [11]. GBM survival under metabolic stress depends on complementary support systems that maintain nutrient access, intracellular recycling, and membrane‐associated signaling [12, 13]. Neoangiogenesis maintains extracellular access to nutrients and oxygen [14], while autophagy provides intracellular metabolic salvage and organelle recycling [12]. In parallel, cholesterol‐enriched membrane microdomains help organize the signaling output of key oncogenic pathways, including phosphoinositide 3‐kinase/protein kinase B (PI3K/AKT) and mitogen‐activated protein kinase (MAPK) [13]. Importantly, GBM cells have been reported to rely strongly on exogenous cholesterol for survival, making cholesterol uptake and intracellular cholesterol handling a therapeutically relevant vulnerability [11, 13]. Lipid structural plasticity can further support adaptation to metabolic stress [15]. Together, these dependencies provide an opportunity to impair overall tumor fitness while simultaneously destabilizing the bioenergetic and signaling support required to maintain stem‐like GBM states.

Regorafenib has undergone clinical evaluation in GBM, providing a translational basis for its inclusion in the present strategy. The randomized phase II REGOMA trial demonstrated clinical activity in recurrent GBM, while the subsequent phase I REGOMA‐2 study extended its evaluation to newly diagnosed O6‐methylguanine‐DNA methyltransferase‐methylated, isocitrate dehydrogenase (IDH)‐wild‐type GBM in combination with temozolomide with or without radiotherapy [16, 17]. Regorafenib‐based studies in GBM have primarily focused on its multikinase and antiangiogenic activities and, more recently, on phosphoserine aminotransferase 1 (PSAT1)‐dependent disruption of autophagic processing [17, 18]. These activities can impose metabolic pressure by restricting extracellular nutrient support and intracellular autophagy‐associated salvage [19], but they do not directly address cholesterol homeostasis, which has emerged as a distinct metabolic dependency in GBM and is particularly relevant to the maintenance of glioma stem‐like states [20, 21]. We therefore selected erythrodiol, a naturally occurring pentacyclic triterpenoid constituent of olive oil that has been investigated in cholesterol efflux and atherosclerosis‐related lipid metabolism [22]. By stabilizing ATP‐binding cassette transporter A1 (ABCA1) and enhancing cholesterol efflux [22, 23], erythrodiol provides a mechanistically complementary means of disrupting cholesterol homeostasis and reducing the membrane cholesterol pool required to maintain signaling‐competent lipid microdomains [24, 25]. We therefore reasoned that simultaneous restriction of extracellular nutrient supply, autophagy‐dependent metabolic salvage, and cholesterol availability could impose convergent bioenergetic and signaling stress on GBM cells. This complementarity further provided the rationale for testing whether regorafenib and erythrodiol interact synergistically under controlled co‐exposure. Such metabolic stress may, in turn, compromise stemness‐supporting programs and promote an immunogenic cell death (ICD)‐associated tumor‐cell state [26], thereby providing a mechanistic link between tumor‐intrinsic metabolic intervention and local immune remodeling [27].

Translating this metabolic pairing to intracranial GBM nevertheless requires coordinated brain‐tumor delivery. Free regorafenib and erythrodiol are constrained by limited and potentially discordant brain exposure, nonspecific biodistribution, and independent pharmacokinetics that can alter the intended drug ratio before both agents reach the same tumor‐cell population [28, 29]. Co‐delivery therefore offers a means of preserving coordinated exposure while coupling the metabolic intervention to brain‐tumor targeting. Among established peptide‐based BBB targeting strategies, Angiopep‐2 predominantly exploits low‐density lipoprotein receptor‐related protein 1 (LRP1)‐mediated transcytosis, whereas apolipoprotein E‐derived ligands can engage multiple members of the low‐density lipoprotein receptor (LDLR) family, including LDLR, LRP1, and low‐density lipoprotein receptor‐related protein 2 [30]. For the present platform, we selected an LDLR‐centered route because LDLR provides a receptor axis relevant to both brain endothelial transport and subsequent GBM‐cell uptake [31, 32]. Apolipoprotein B (ApoB)‐derived LDLR‐binding sequences have previously been shown to support BBB transport of macromolecular cargo [33]. To implement this route without reconstructing a native low‐density lipoprotein (LDL) particle, we used a recombinant human ApoB fragment spanning His3365‐Glu3548, which contains the canonical LDLR‐recognition region [34]. Because full‐length ApoB100 comprises 4536 amino acids and exceeds 500 kDa [35], the defined ApoB fragment provides a more practical interfacial ligand for nanoparticle functionalization while retaining an LDLR‐recognition interface capable of supporting receptor‐mediated brain‐tumor delivery [36].

Here, we developed apolipoprotein B fragment‐decorated, regorafenib/erythrodiol‐loaded nanoparticles (ApoBf‐R/E@NPs) as an LDLR‐oriented biomimetic nanoplatform that couples brain‐tumor delivery with coordinated metabolic intervention. The formulation co‐encapsulates regorafenib and erythrodiol to preserve their combined exposure while imposing complementary constraints on autophagy‐associated nutrient salvage and cholesterol‐dependent metabolic support. We show that the two agents exhibit a synergistic pharmacological interaction and that their coordinated delivery produces convergent bioenergetic and signaling stress in GBM cells. This metabolic pressure broadly suppresses GBM cell survival and proliferative capacity while concurrently weakening stemness‐associated programs and reducing the viability and sphere‐forming capacity of stem‐like GBM cells. In parallel, treatment promotes an ICD‐associated tumor‐cell state and is accompanied by remodeling of antitumor immunity. Across immunocompetent models, these effects translate into control of orthotopic tumor growth, inhibition of untreated distal tumor progression, and delayed regrowth after subtotal resection. Together, these findings establish a therapeutic framework in which BBB‐oriented nanodelivery enables a complementary metabolic intervention that acts broadly against GBM, retains functional activity against the stem‐like compartment, and promotes antitumor immune responses.

## 2 Results and Discussion

### 2.1 Design, Synthesis, and Physicochemical Characterization of ApoBf‐R/E@NPs

To translate this rationale into a deliverable formulation, we designed ApoBf‐R/E@NPs as a lipid‐based, LDLR‐targeted delivery system rather than as a reconstituted LDL particle. The design was intended to retain the transport logic of LDLR engagement while keeping the formulation chemically simpler and more reproducible than full ApoB‐based LDL mimics. The formulation relies on hydrophobic co‐assembly of regorafenib and erythrodiol into a therapeutic core, steric stabilization by an amphiphilic 1,2‐Distearoyl‐sn‐Glycero‐3‐Phosphoethanolamine (DSPE) conjugated Polyethylene Glycol (DSPE‐PEG) shell, and post‐assembly surface decoration with LDLR‐targeting ApoB‐frags through cooperative electrostatic and supramolecular interactions (Figure 1A). As the colloidal stability and targeting performance of the engineered interface depend on the protein‐to‐lipid balance [37], varying concentrations of ApoB fragments were evaluated by monitoring changes in hydrodynamic diameter and zeta potential. At an ApoB‐frag input of 2.0 µg/mg, the surface charge had approached the mildly positive plateau while the particle diameter remained close to 160 nm; further increases in ApoB‐frag input produced only modest additional changes in zeta potential but a more pronounced increase in particle size; this ratio was therefore selected for subsequent experiments (Figure S1A,B). Having established the interfacial composition, we next examined whether the resulting nanoplatform displayed the expected morphology and physicochemical features. Morphological analysis by transmission electron microscopy (TEM) and scanning electron microscopy (SEM) showed that ApoBf‐R/E@NPs exhibited a uniform spherical architecture with a well‐defined nanoscale morphology (Figure 1B,C). Dynamic light scattering (DLS) measurements showed a mean hydrodynamic diameter of 165.7 nm with a narrow polydispersity index (PDI < 0.20) and a pronounced Tyndall effect (Figure 1D). The unmodified R/E@NPs displayed a diameter of 141.2 nm (Figure S1C); this increase reflects the additional volume contributed by the interfacial peptide layer. X‐ray photoelectron spectroscopy (XPS) revealed the expected elemental signatures of the assembled formulation (Figure 1E). Energy‐dispersive X‐ray spectroscopy (EDS) mapping confirmed the homogeneous distribution of carbon (C), oxygen (O), nitrogen (N), phosphorus (P), and fluorine (F) within the nanostructure, corroborating the integration of the therapeutic payloads and lipid components (Figure 1F).

**FIGURE 1:**
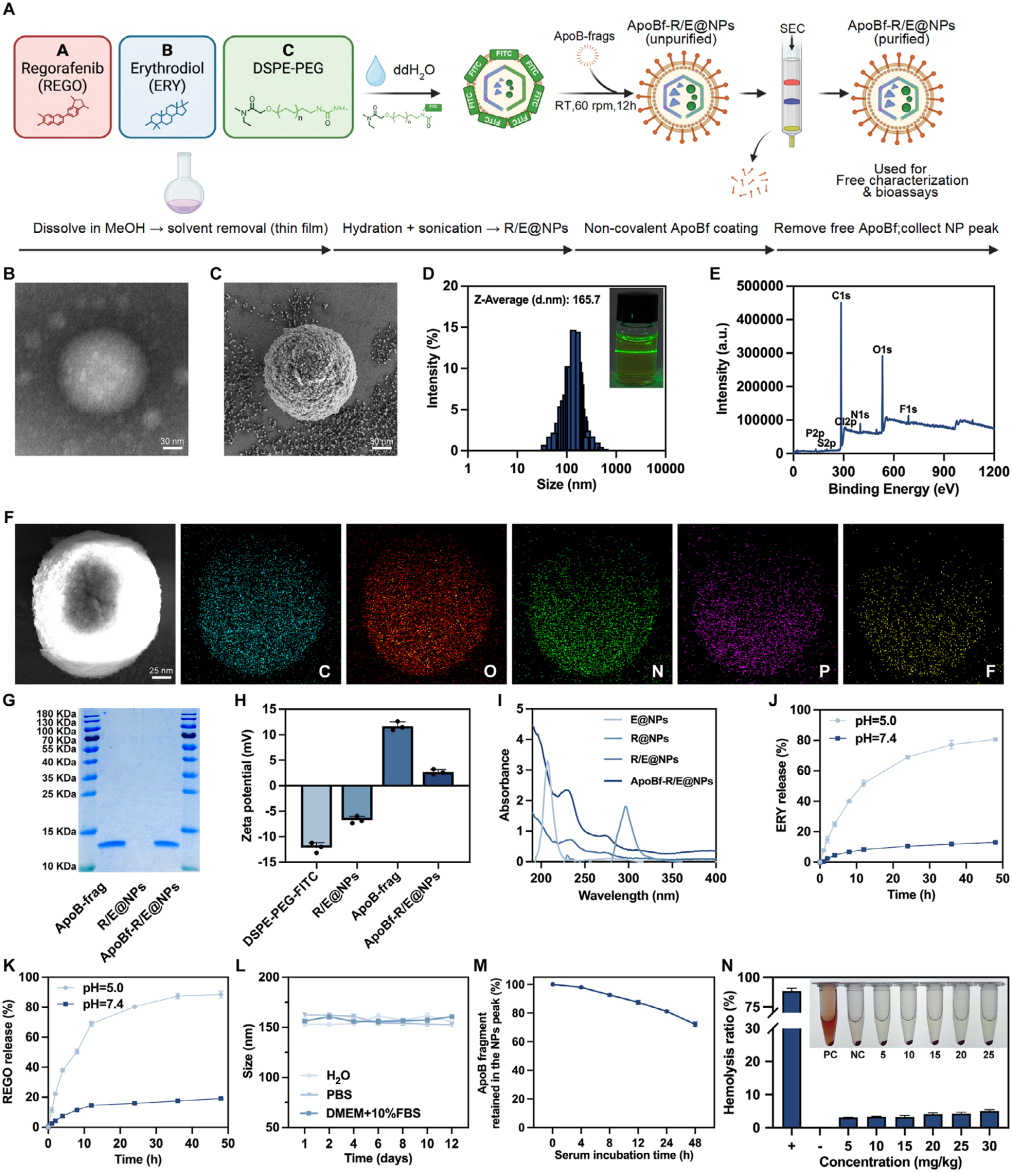
Preparation and characterization of ApoBf‐R/E@NPs. (A) Schematic illustration of the ApoBf‐R/E@NPs formulation process, including dual‐drug co‐assembly via thin‐film hydration, subsequent biointerfacial decoration with ApoB‐frags, and final isolation by SEC. (B,C) Representative TEM (B) and SEM (C) images of ApoBf‐R/E@NPs. (D) Hydrodynamic size distribution of ApoBf‐R/E@NPs measured by DLS. Inset: macroscopic visualization of the Tyndall effect. (E) XPS survey spectrum confirming the elemental composition. (F) EDS mapping resolving the homogeneous spatial distribution of C, O, N, P, and F. (G) Sodium dodecyl sulfate‐polyacrylamide gel electrophoresis (SDS‐PAGE) coupled with Coomassie Brilliant Blue staining supporting the retention and surface association of ApoB‐frags on the nanoparticles. (H) Zeta potential profiles of DSPE‐PEG‐FITC, bare R/E@NPs, free ApoB‐frags, and fully assembled ApoBf‐R/E@NPs, supporting amphipathic interfacial anchoring of the ApoB‐frags. (I) Ultraviolet‐visible (UV‐vis) absorption spectra showing dual‐drug co‐encapsulation, as evidenced by the superposition of the characteristic peaks of erythrodiol and regorafenib. (J,K) pH‐responsive in vitro release profiles of (J) erythrodiol and (K) regorafenib from ApoBf‐R/E@NPs at pH 5.0 and 7.4 over 48 h. (L) Colloidal stability monitored by DLS in different media (water, PBS, and DMEM supplemented with 10% FBS) over 12 days. (M) Quantitative assessment of ApoB‐frags detachment over 48 h. (N) Hemolysis assay evaluating the hemocompatibility of ApoBf‐R/E@NPs at increasing concentrations. PC, positive control; NC, negative control.

We next asked whether the targeting interface was stably assembled and whether both drugs were successfully co‐encapsulated. To verify that the protein was anchored via stable supramolecular interactions rather than transient physical mixing, size‐exclusion chromatography (SEC) was performed. The fluorescent lipid signal (fluorescein isothiocyanate, FITC) and the protein signal (A280) co‐eluted in early void‐volume fractions, whereas free ApoB fragments showed delayed elution due to size‐exclusion partitioning, consistent with successful interfacial assembly (Figure S1D‐G). Coomassie Brilliant Blue staining further confirmed the retention of ApoB‐frags in the purified nanoparticle fraction (Figure 1G). Together, these results support the stable association and functional accessibility of ApoB‐frags on the assembled nanoparticles. Microelectrophoretic analysis showed that the zeta potential shifted from ‐6.7 mV for R/E@NPs to +2.7 mV for ApoBf‐R/E@NPs, intermediate between the negatively charged lipid formulation and free ApoB‐frags (+11.7 mV) (Figure 1H). UV‐Vis spectroscopy further showed that ApoBf‐R/E@NPs retained spectral features attributable to both erythrodiol‐ and regorafenib‐containing formulations (Figure 1I). Using matrix‐matched high‐Performance liquid chromatography (HPLC) calibration curves for the two agents (Figure S1H‐K), the encapsulation efficiencies were determined to be 89.5 ± 2.4% for regorafenib and 91.2 ± 1.8% for erythrodiol, with a total drug loading capacity of 13.8 ± 1.1%. The high incorporation efficiencies are consistent with the hydrophobic interactions and noncovalent molecular packing that stabilize lipid‐based co‐assemblies of poorly water‐soluble compounds [38].

### 2.2 pH‐Responsive Release, Colloidal Stability, and Hemocompatibility

We next examined the release behavior of the co‐loaded formulation under neutral and acidic conditions corresponding to systemic and endolysosomal environments, respectively. At pH 7.4, cumulative release over 48 h remained limited to 19.1% for regorafenib and 13.0% for erythrodiol. In contrast, release was markedly accelerated at pH 5.0, reaching 88.5% for regorafenib and 80.6% for erythrodiol over the same period (Figure 1J,K). This pH‐dependent behavior is unlikely to reflect only dissociation of surface‐associated biomolecules [39], but is more plausibly related to reduced stability of the noncovalently co‐assembled drug‐lipid matrix under acidic conditions [40]. Because regorafenib is only weakly basic and is expected to remain largely unprotonated at pH 5.0, direct drug protonation is unlikely to be the sole determinant of the accelerated release. Rather, an acidic endolysosomal‐like environment may perturb noncovalent interactions within the assembly [41, 42], weaken the intermolecular forces contributing to DSPE‐PEG‐based structural cohesion [43], and thereby facilitate payload liberation from the nanocarrier [44]. Thus, the formulation favors payload retention under neutral conditions while permitting substantially greater release in an acidic intracellular environment.

Beyond drug release, we assessed whether the assembled formulation maintained its physicochemical integrity under conditions relevant to subsequent biological studies. ApoBf‐R/E@NPs exhibited little change in hydrodynamic size in water, phosphate‐buffered saline (PBS), or Dulbecco's modified Eagle medium (DMEM) supplemented with 10% fetal bovine serum (FBS) over 12 days, with no evident macroscopic aggregation (Figure 1L). ApoB‐frag association declined gradually during serum incubation but remained above approximately 70% at 48 h (Figure 1M), indicating substantial retention of the targeting interface over the relevant experimental window. Hemolysis remained below 5% across the tested concentration range (Figure 1N), supporting favorable compatibility with erythrocytes. Together, these findings establish a co‐loaded ApoBf‐functionalized formulation with stable nanoscale characteristics, substantial ligand retention, pH‐dependent payload release, and favorable hemocompatibility, providing the material foundation for the LDLR‐dependent targeting and biological studies described below.

### 2.3 LDLR Expression Across Heterogeneous GBM Supports ApoBf‐Mediated Targeted Delivery

Given that ApoBf‐mediated delivery relies on LDLR recognition, we first characterized LDLR expression across heterogeneous GBM contexts using independent transcriptomic datasets. LDLR expression was higher in GBM than in normal brain tissues (Figure 2A), and an independent dataset likewise showed elevated LDLR expression in GSCs relative to normal brain (Figure 2B). Spatial profiling using the Ivy Glioblastoma Atlas Project (IGAP) showed that LDLR was detectable across major anatomical regions of GBM, with lower expression toward the leading edge and infiltrating tumor and broader expression within cellular, perinecrotic, and vascular‐associated regions (Figure 2C). LDLR was also retained across molecular subtypes, with relatively higher distributions in classical/mesenchymal tumors than in neural/proneural tumors in the CGGA cohort [45]; a similar subtype‐dependent pattern was observed in the independent Wang‐classified subtype [46] (Figure 2D and Figure S2A). At the malignant‐cell‐state level [47], AC‐like and MES‐like populations showed greater LDLR expression than NPC‐like and OC‐like states (Figure 2E). Thus, despite substantial inter‐ and intratumoral heterogeneity, LDLR remained represented across the major GBM contexts relevant to ApoBf‐mediated targeting, including the stem‐like compartment.

**FIGURE 2:**
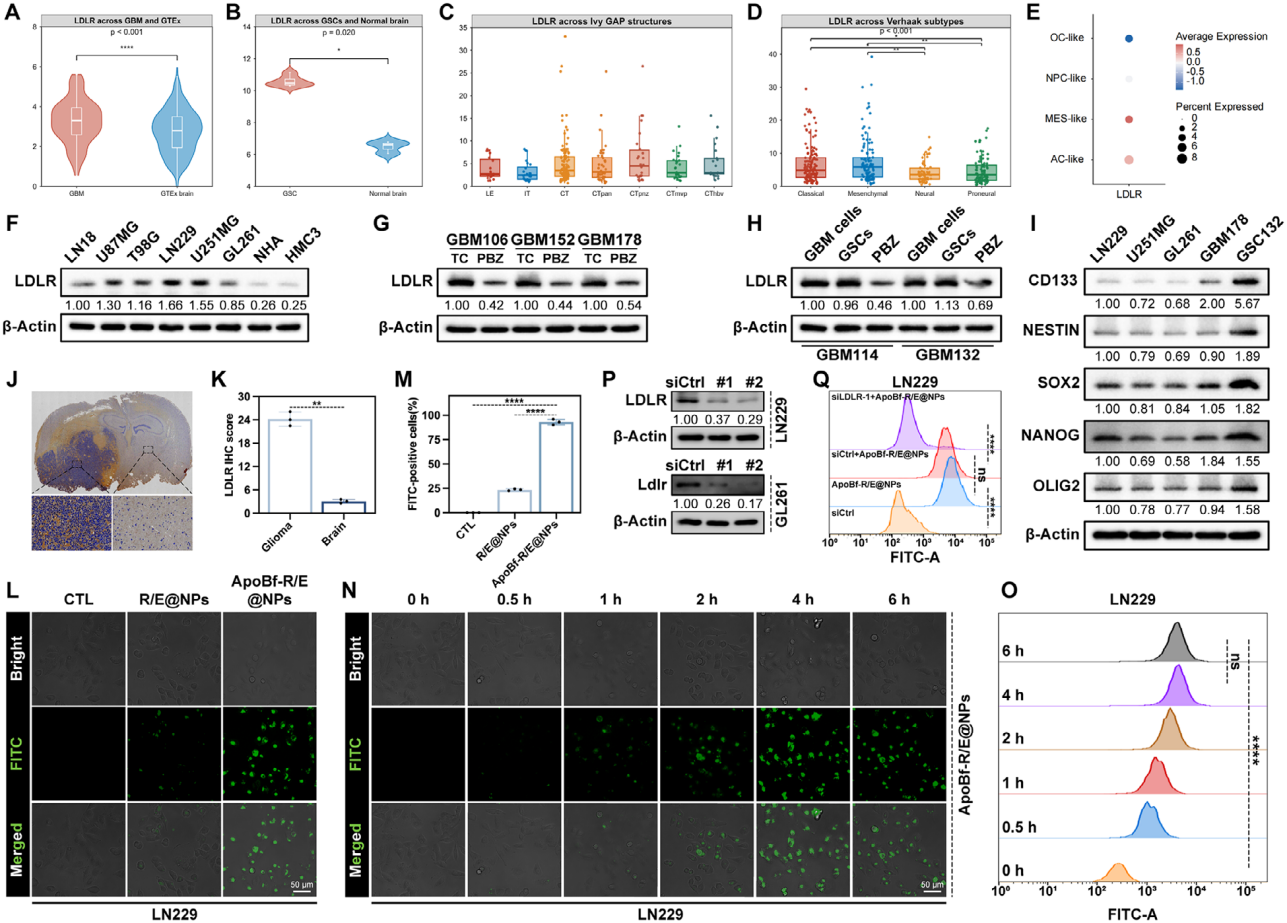
LDLR expression across heterogeneous GBM supports ApoBf‐mediated targeted delivery. (A) LDLR expression in TCGA‐GBM and GTEx normal brain tissues. (B) LDLR expression in GSCs and normal brain in an independent transcriptomic dataset. (C) LDLR expression across Ivy Glioblastoma Atlas Project (IGAP) anatomical compartments, including leading edge (LE), infiltrating tumor (IT), cellular tumor (CT), pseudopalisading cells around necrosis (PAN), perinecrotic zone (PNZ), microvascular proliferation (MVP), and hyperplastic blood vessels (HBV). (D) LDLR expression across Verhaak‐defined classical, mesenchymal, neural, and proneural GBM transcriptional subtypes. (E) Dot‐plot representation of LDLR expression across AC‐like, MES‐like, NPC‐like, and OC‐like malignant cell states; dot size denotes the percentage of LDLR‐expressing cells and color denotes average expression. (F) Representative immunoblot analysis of LDLR in established GBM cell lines, normal human astrocytes (NHA), and HMC3 microglial cells. (G) LDLR immunoblotting in paired tumor‐core (TC) and peritumoral brain‐zone (PBZ) specimens from GBM106, GBM152, and GBM178. (H) LDLR immunoblotting in patient‐derived GBM cells, corresponding GSC cultures, and PBZ samples. (I) Immunoblot analysis of CD133, NESTIN, SOX2, NANOG, and OLIG2 in established GBM cells and patient‐derived GBM/GSC cultures. (J,K) Representative LDLR immunohistochemistry (IHC) of orthotopic GL261 glioma and spatially distant non‐tumor brain tissue, with enlarged views of the boxed regions (J), and corresponding quantitative scoring (K). (L,M) Uptake of R/E@NPs and ApoBf‐R/E@NPs in LN229 cells assessed by fluorescence imaging (L) and quantification of formulation‐associated FITC‐positive cells normalized to total cell number (M). Scale bar, 50 µm. (N,O) Time‐dependent uptake of ApoBf‐R/E@NPs in LN229 cells over 0–6 h assessed by fluorescence imaging (N) and flow cytometry with corresponding quantitative analysis (O). Scale bar, 50 µm. (P) Immunoblot validation of siRNA‐mediated LDLR/Ldlr knockdown in LN229 and GL261 cells. (Q) Flow‐cytometric analysis and corresponding quantification of ApoBf‐R/E@NPs‐associated fluorescence in control and LDLR‐silenced LN229 cells.

We next examined LDLR abundance in experimental GBM models and patient‐derived material. Representative immunoblotting showed variable LDLR abundance across established GBM cell lines, whereas comparatively weaker signals were detected in normal human astrocytes (NHA) and HMC3 human microglial cells under the same assay conditions (Figure 2F). In paired patient specimens, tumor cores showed higher LDLR abundance than the corresponding peritumoral brain zones (PBZs) (Figure 2G). LDLR was also readily detectable in patient‐derived primary GBM cells and GSC cultures, with lower abundance in the corresponding PBZ samples (Figure 2H). The stem‐like phenotype of the GSC cultures was supported by CD133, NESTIN, SOX2, NANOG, and OLIG2 expression (Figure 2I). In the orthotopic GL261 model, LDLR immunohistochemistry (IHC) showed substantially stronger staining within glioma tissue than in spatially distant non‐tumor brain tissue, a difference further supported by quantitative scoring (Figure 2J,K). Taken together, these findings establish that LDLR is retained across established GBM models, patient‐derived tumor cells, stem‐like cultures, and orthotopic tumors, while showing relative enrichment over non‐tumor comparators in several experimental and clinical contexts.

We then asked whether this receptor distribution translated into ApoBf‐dependent cellular delivery. In LN229 cells, ApoBf‐R/E@NPs showed greater intracellular accumulation than non‐targeted R/E@NPs, as visualized by fluorescence imaging and supported by cell‐normalized quantification of formulation‐associated FITC‐positive cells (Figure 2L,M). A similar targeting advantage was observed in GL261 cells (Figure S2B,C). Time‐dependent fluorescence imaging and flow cytometry showed progressive uptake of ApoBf‐R/E@NPs over the first several hours, reaching its highest level at approximately 4 h in LN229 cells and showing a comparable temporal pattern in GL261 cells (Figure 2N,O and Figure S2D,E). LDLR knockdown was verified by immunoblotting in both cell lines (Figure 2P), and LDLR silencing markedly shifted ApoBf‐R/E@NP‐associated fluorescence toward lower levels (Figure 2Q and Figure S2F). These data establish a functional contribution of LDLR to ApoBf‐mediated cellular internalization and provide the receptor‐level basis for the targeting advantage evaluated in subsequent therapeutic studies.

### 2.4 ApoBf‐R/E@NPs Facilitate BBB‐Crossing Delivery and Preferential Antitumor Activity

Having confirmed LDLR‐dependent internalization, we next investigated whether this targeting advantage translated into enhanced antitumor efficacy. The in vitro therapeutic activity of ApoBf‐R/E@NPs was first evaluated across a panel of human and murine glioma models, including established human GBM cell lines, patient‐derived GBM cells (GBM106), and murine GL261 glioma cells. Dual‐drug formulations generally produced greater viability loss than the corresponding single‐agent formulations, whereas the additional benefit conferred by ApoBf functionalization varied across cell models, consistent with the heterogeneous LDLR expression characterized above (Figure 3A‐E; Figure S3A,B). In contrast, NHA and HMC3 exhibited substantially lower sensitivity to ApoBf‐R/E@NPs under equivalent treatment conditions (Figure S3C,D), supporting preferential cytotoxicity toward malignant glioma cells. Because formulation‐level comparisons cannot distinguish an intrinsic drug interaction from delivery‐related effects, we next examined free regorafenib and free erythrodiol using fixed‐ratio combination‐index (CI) analysis. In LN229 cells, CI values remained below 1 across the tested concentration range, with the interaction becoming more pronounced from low to intermediate concentrations and reaching its lowest CI at 2 µM (Figure S3E). Importantly, a comparable CI profile was reproduced in patient‐derived GBM106 cells (Figure 3F), supporting a synergistic pharmacological interaction between regorafenib and erythrodiol under the tested 1:1 molar ratio and concentration range. These data define the intrinsic interaction between regorafenib and erythrodiol. To distinguish this pharmacological effect from the delivery contribution of ApoBf functionalization, we next evaluated the formulation in an in vitro BBB model that directly interrogates transendothelial transport.

**FIGURE 3:**
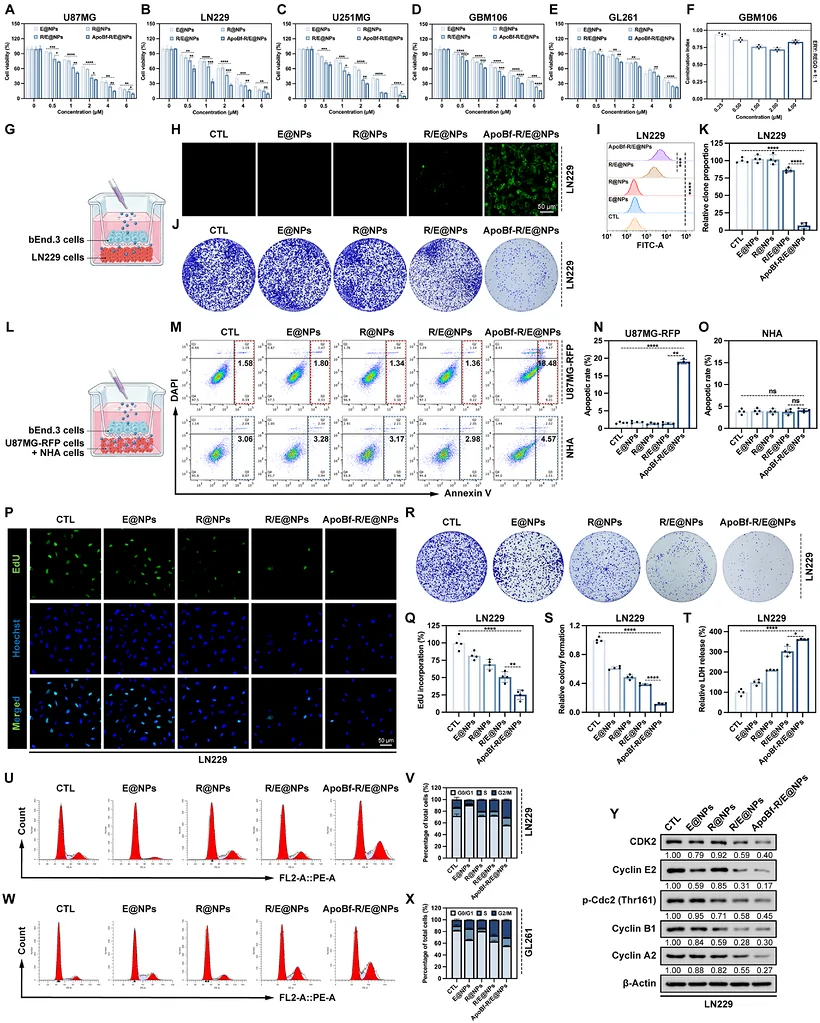
ApoBf‐R/E@NPs facilitate BBB‐crossing delivery and preferential antitumor activity against GBM. (A‐E) Cell viability assessed by CCK‐8 assay in U87MG (A), LN229 (B), U251MG (C), patient‐derived GBM106 cells (D), and murine GL261 glioma cells (E) following treatment with the indicated formulations. (F) Fixed‐ratio combination‐index (CI) analysis of free regorafenib and free erythrodiol in patient‐derived GBM106 cells at a 1:1 molar ratio over the indicated concentration range. The dashed line denotes CI = 1; CI < 1 indicates synergism, CI = 1 additivity, and CI > 1 antagonism. (G) Schematic of the in vitro BBB transwell model with bEnd.3 endothelial cells in the upper chamber and LN229 cells in the lower chamber. (H,I) Transendothelial formulation delivery assessed by fluorescence imaging (H) and flow cytometry with corresponding quantitative analysis (I) in recipient LN229 cells. Scale bar, 50 µm. (J,K) Clonogenic survival of LN229 cells following transendothelial delivery (J) and corresponding quantification (K). (L) Schematic of the BBB‐associated tumor‐normal cell co‐culture model comprising bEnd.3 endothelial cells, U87MG‐RFP cells, and NHA cells. (M) Representative Annexin V/DAPI flow‐cytometric profiles of separately gated U87MG‐RFP and NHA populations following treatment with the indicated formulations. (N,O) Quantification of apoptotic U87MG‐RFP cells (N) and NHA cells (O). (P,Q) Representative EdU incorporation images in LN229 cells (P) and quantification of EdU‐positive cells (Q). Scale bar, 50 µm. (R,S) Clonogenic survival assay in LN229 cells (R) and corresponding colony quantification (S). (T) LDH release from LN229 cells following treatment with the indicated formulations. (U,V) Flow‐cytometric analysis of cell‐cycle distribution in LN229 cells (U) and corresponding quantification (V). (W,X) Flow‐cytometric analysis of cell‐cycle distribution in GL261 cells (W) and corresponding quantification (X). (Y) Immunoblot analysis of CDK2, Cyclin E2, p‐cdc2 (Thr161), Cyclin B1, and Cyclin A2 in LN229 cells.

An in vitro BBB transwell model was established using bEnd.3 endothelial cells in the upper chamber and LN229 cells in the lower chamber (Figure 3G). ApoBf‐R/E@NPs exhibited enhanced transendothelial transport compared with non‐targeted counterparts, as demonstrated by fluorescence imaging and flow cytometric analysis of recipient GBM cells (Figure 3H,I). Consistent with enhanced BBB traversal, ApoBf‐R/E@NPs significantly reduced clonogenic survival of GBM cells in the lower chamber (Figure 3J,K). Similar results were obtained in the GL261‐based BBB model (Figure S3F–J). To evaluate tumor selectivity within a more complex cellular context, BBB‐associated tumor‐normal cell co‐culture models were established by combining bEnd.3 endothelial cells with U87MG‐red fluorescent protein (RFP) cells and either NHA or HMC3 cells (Figure 3L and Figure S3K). Tumor cells were distinguished from normal brain‐associated cells based on RFP fluorescence, enabling independent quantification of apoptotic responses in each population. In the U87MG‐RFP/NHA co‐culture model, ApoBf‐R/E@NPs preferentially induced apoptosis in RFP‐positive tumor cells while exerting limited effects on NHA cells (Figure 3M–O). A similar pattern was observed in the U87MG‐RFP/HMC3 model, where ApoBf‐R/E@NPs triggered stronger apoptotic responses in GBM cells than in microglial cells (Figure S3L‐N).

The antitumor activity of ApoBf‐R/E@NPs was further reflected by their effects on proliferative capacity and cell‐cycle progression. ApoBf‐R/E@NP treatment markedly reduced 5‐ethynyl‐2’‐deoxyuridine (EdU) incorporation (Figure 3P,Q and Figure S3O,P) and clonogenic survival (Figure 3R,S and Figure S3Q,R) in LN229 and GL261 cells, indicating sustained suppression of DNA synthesis and long‐term proliferative potential. Consistently, lactate dehydrogenase (LDH) release assays revealed increased membrane damage following ApoBf‐R/E@NP treatment (Figure 3T; Figure S3S). Flow cytometric analysis showed that E@NPs and R@NPs produced limited cell‐cycle perturbation, whereas ApoBf‐R/E@NPs induced pronounced G2/M arrest (Figure 3U–X). This arrest was accompanied by reduced expression of G1/S‐associated regulators (CDK2 and Cyclin E2) and G2/M‐associated regulators (p‐cdc2, Cyclin B1, and Cyclin A2) (Figure 3Y; Figure S3T). Together, these results demonstrate that ApoBf functionalization enhances BBB‐crossing delivery and confers preferential antitumor activity against GBM cells.

### 2.5 ApoBf‐R/E@NPs Induce Autophagic‐Lysosomal Dysfunction and Suppress Angiogenesis

To further elucidate the mechanistic basis of the R/E interaction, we investigated the autophagy‐lysosome axis, which serves as a critical metabolic adaptation pathway sustaining GBM survival under nutrient stress [15]. Confocal microscopy revealed that ApoBf‐R/E@NP treatment induced the accumulation of disorganized LC3B puncta accompanied by reduced colocalization with the lysosomal marker LAMP1, indicating impaired autophagosome‐lysosome fusion (Figure 4A,B and Figure S4A,B). To distinguish enhanced autophagosome generation from defective downstream degradation, a monomeric red fluorescent protein‐green fluorescent protein‐LC3 (mRFP‐GFP‐LC3) tandem reporter system was employed. Under basal conditions, control cells predominantly exhibited red puncta (mRFP^+^/GFP^−^), reflecting efficient autolysosomal maturation. In contrast, ApoBf‐R/E@NP‐treated cells displayed increased yellow puncta (mRFP^+^/GFP^+^) together with residual red puncta, indicating autophagosome accumulation and impaired autophagic flux rather than complete blockade of autophagosome formation (Figure 4C,D and Figure S4C,D). Consistently, reduced dye‐quenched bovine serum albumin (DQ‐BSA) fluorescence further demonstrated compromised lysosomal degradative activity following ApoBf‐R/E@NP treatment (Figure 4E,F and Figure S4E,F).

**FIGURE 4:**
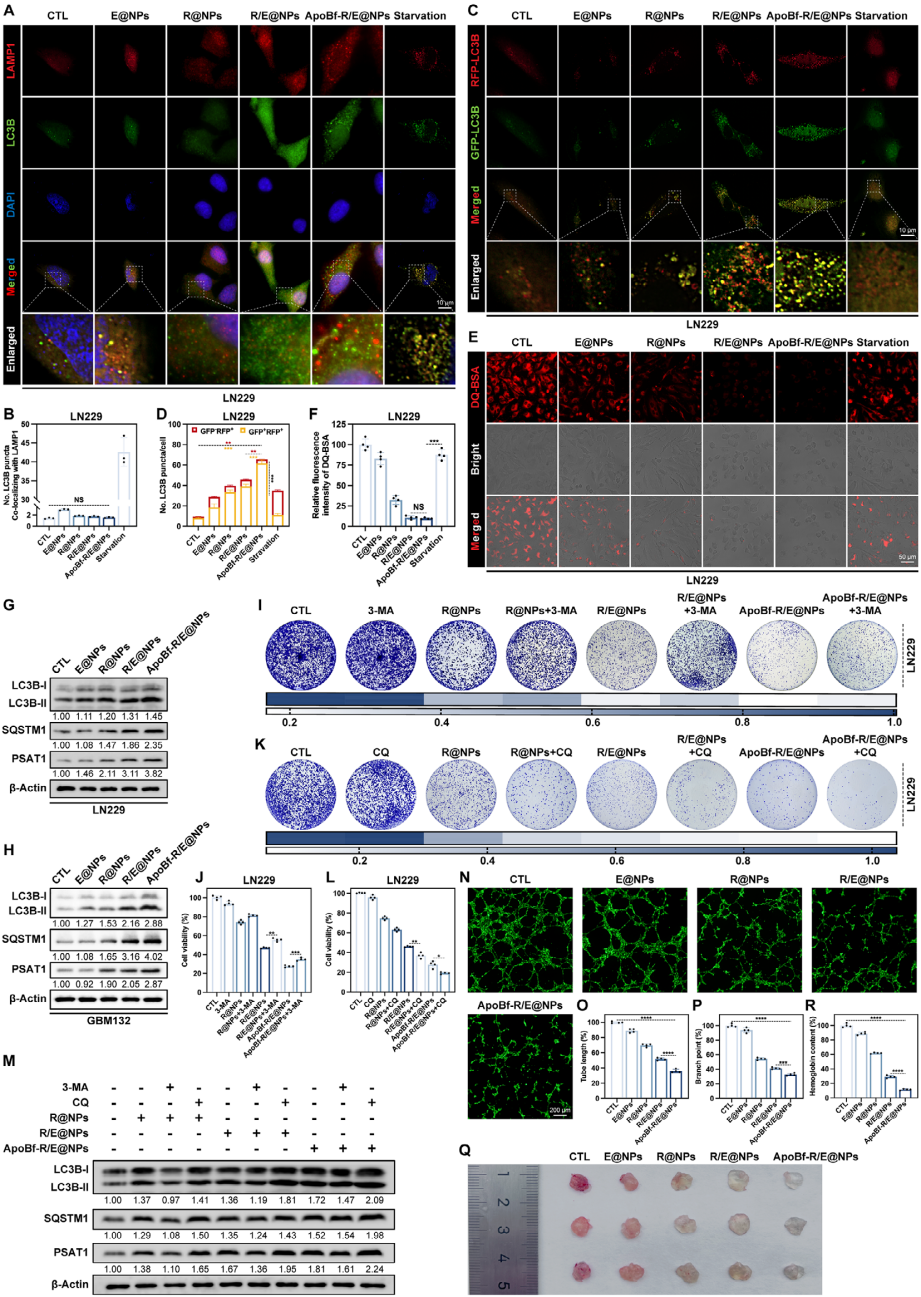
ApoBf‐R/E@NPs impair autophagic‐lysosomal function and angiogenesis. (A,B) Confocal imaging with enlarged views of the boxed regions (A) and colocalization analysis (B) of LC3B and LAMP1 in LN229 cells. (C, D) Evaluation of autophagic flux using the mRFP‐GFP‐LC3 tandem reporter, including representative images with enlarged views of the boxed regions (C) and puncta quantification (D). (E, F) Assessment of lysosomal proteolytic activity via DQ‐BSA fluorescence (E) and intensity quantification (F). (G, H) Immunoblotting of autophagic markers (LC3B, SQSTM1/p62) and PSAT1 in LN229 (G) and GBM132 (H) cells. (I, J) Clonogenic survival and cell viability following treatment with the early‐stage autophagy inhibitor 3‐MA. The horizontal color scale beneath the colony images indicates normalized colony density (0.2–1.0), with values normalized to the control group (CTL = 1.0); darker blue represents higher colony density. (K, L) Survival and cell viability following treatment with the late‐stage lysosomal inhibitor CQ. (M) Immunoblot analysis of LC3B, SQSTM1, and PSAT1 accumulation following 3‐MA or CQ treatment in LN229 cells. (N‐P) Antiangiogenic activity of ApoBf‐R/E@NPs in HUVEC tube formation assays, including representative images (N), quantification of total tube length (O), and branching points (P). (Q, R) In vivo antiangiogenic efficacy evaluated by a subcutaneous Matrigel plug assay, showing representative explanted plugs (Q) and quantitative hemoglobin content (R).

Regorafenib has been reported to interfere with autophagic processing through stabilization of phosphoserine aminotransferase 1 (PSAT1) [19]. We therefore examined whether ApoBf‐mediated co‐delivery preserved this pharmacological vulnerability. Immunoblotting revealed marked accumulation of LC3B‐II, SQSTM1/p62, and PSAT1 following ApoBf‐R/E@NP treatment in LN229 and GL261 cells (Figure 4G; Figure S4G), consistent with disrupted autophagic processing. Importantly, patient‐derived GBM132 cells exhibited similar accumulation of LC3B‐II, SQSTM1/p62, and PSAT1 after ApoBf‐R/E@NP treatment (Figure 4H), indicating that the autophagic‐lysosomal dysfunction induced by the nanoplatform is also present in clinically relevant GBM models. To define the contribution of autophagic dysfunction to ApoBf‐R/E@NP‐mediated cytotoxicity, pharmacological complementation experiments were performed. Inhibition of early‐stage autophagy initiation by 3‐methyladenine (3‐MA) partially rescued cell viability and reduced the accumulation of LC3B‐II, SQSTM1/p62, and PSAT1 (Figure 4I,J,L and Figure S4H,I,L). In contrast, chloroquine (CQ)‐mediated inhibition of lysosomal degradation further enhanced cytotoxicity and promoted accumulation of autophagy‐associated proteins (Figure 4K‐M and Figure S4J‐L). The distinct responses to 3‐MA and CQ suggest that ApoBf‐R/E@NPs do not completely abolish lysosomal function but instead create a functional imbalance between sustained upstream autophagosome formation and insufficient downstream clearance. Under this condition, GBM cells remain dependent on residual lysosomal activity to maintain metabolic homeostasis, and further disruption of this compensatory capacity by CQ aggravates organelle stress [42, 48]. Because the autophagy‐lysosome continuum functions as a major nutrient salvage pathway that supports mitochondrial oxidative phosphorylation and metabolic plasticity in GBM cells [49], its disruption represents an important vulnerability exploited by ApoBf‐R/E@NPs.

We next investigated whether the formulation retained the antiangiogenic activity of regorafenib by limiting extracellular metabolic support. Given the dependence of GBM progression on vascular remodeling and neovascularization [50], the effects of ApoBf‐R/E@NPs on endothelial angiogenic behavior were evaluated. In vitro, ApoBf‐R/E@NPs treatment markedly impaired human umbilical vein endothelial cells (HUVECs) tube formation, resulting in reduced total tube length and branching points compared with control formulations (Figure 4N–P). Consistently, Matrigel plug assays demonstrated reduced vascularization following ApoBf‐R/E@NPs treatment, as evidenced by reduced gross vascularization and lower hemoglobin content within the plugs (Figure 4Q,R). Together, these findings indicate that ApoBf‐R/E@NPs simultaneously disrupt intracellular nutrient recycling through autophagic‐lysosomal dysfunction and restrict extracellular metabolic support through antiangiogenic activity, thereby establishing a dual metabolic constraint on GBM progression.

### 2.6 ApoBf‐R/E@NPs Drive Bioenergetic Collapse Via Autophagic Dysfunction and ABCA1‐Associated Lipid Depletion

Disruption of autophagic nutrient recycling imposes substantial stress on mitochondrial homeostasis, ultimately compromising cellular bioenergetic capacity [51]. Fluorescence imaging and flow cytometric analysis revealed elevated intracellular reactive oxygen species (ROS) following ApoBf‐R/E@NP treatment (Figure 5A,B and Figure S5A,B). This oxidative stress was accompanied by a marked reduction in mitochondrial membrane potential (ΔΨm), as indicated by the transition from JC‐1 aggregates to monomers (Figure 5C,D and Figure S5C,D). Loss of mitochondrial integrity is closely associated with activation of the intrinsic apoptotic pathway [52]. Consistently, dual‐staining flow cytometry demonstrated increased apoptosis following treatment with ApoBf‐R/E@NPs (Figure 5E,F and Figure S5E,F). Importantly, patient‐derived GBM132 cells exhibited a comparable increase in apoptotic populations after ApoBf‐R/E@NP treatment (Figure S5G,H), extending the observed cytotoxic response beyond established GBM cell lines. Immunoblotting further confirmed activation of mitochondrial apoptosis, as evidenced by increased Bax expression, reduced Bcl‐2 levels, and enhanced cleavage of Caspase‐3 and PARP‐1 (Figure 5G and Figure S5I). Similar apoptotic molecular alterations were also detected in patient‐derived GBM132 cells (Figure S5J), further supporting the reproducibility of ApoBf‐R/E@NP‐induced apoptosis in clinically relevant GBM models. These findings indicate that ApoBf‐R/E@NPs effectively convert metabolic stress induced by autophagic dysfunction into mitochondria‐dependent apoptotic cell death.

**FIGURE 5:**
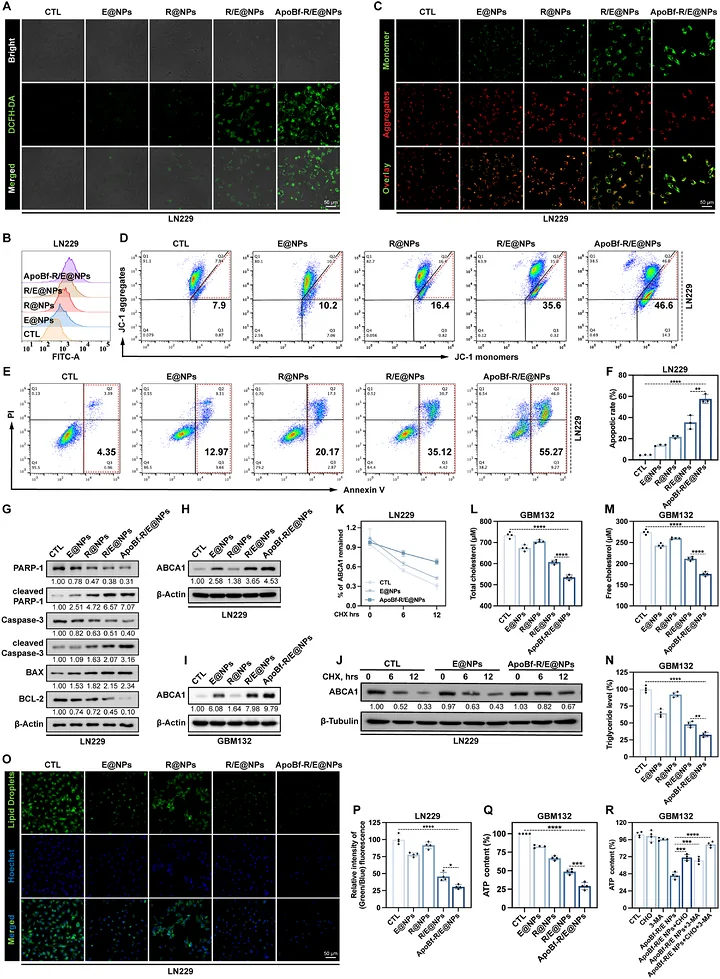
ApoBf‐R/E@NPs induce mitochondrial dysfunction, ABCA1 stabilization, and bioenergetic collapse in GBM cells. (A, B) Intracellular ROS in LN229 cells following treatment with the indicated formulations, assessed by 2’,7’‐dichlorodihydrofluorescein diacetate (DCFH‐DA) fluorescence imaging (A) and flow cytometry (B). Scale bar, 50 µm. (C, D) Mitochondrial membrane potential (ΔΨm) in LN229 cells assessed by JC‐1 fluorescence imaging (C) and flow cytometry (D). Scale bar, 50 µm. (E, F) Annexin V/PI flow‐cytometric analysis of apoptosis in LN229 cells (E) and corresponding quantification (F). (G) Immunoblot analysis of apoptosis‐associated proteins, including Bax, Bcl‐2, Caspase‐3, cleaved Caspase‐3, PARP‐1, and cleaved PARP‐1, in LN229 cells. (H, I) Immunoblot analysis of ABCA1 abundance in LN229 cells (H) and patient‐derived GBM132 cells (I). (J, K) Cycloheximide (CHX) chase analysis of ABCA1 stability in LN229 cells, showing representative immunoblots at 0, 6, and 12 h (J) and quantification of the remaining ABCA1 protein (K). (L–N) Biochemical quantification of total cholesterol (L), free cholesterol (M), and triglycerides (N) in patient‐derived GBM132 cells using the corresponding assay kits. (O, P) Fluorescence imaging of intracellular lipid droplets in LN229 cells (O) and quantitative analysis of fluorescence intensity (P). Scale bar, 50 µm. (Q) Intracellular ATP levels in patient‐derived GBM132 cells following treatment with the indicated formulations. (R) Functional complementation of ATP levels in GBM132 cells following cholesterol supplementation (+CHO), inhibition of autophagy initiation with 3‐MA (+3‐MA), or their combination (+CHO+3‐MA).

This bioenergetic collapse likely arises from the combined disruption of intracellular recycling and lipid metabolic homeostasis. GBM cells rely heavily on cholesterol and lipid metabolism to support membrane biogenesis, signaling, and rapid proliferation [20]. Beyond impairing autophagic nutrient salvage, we therefore examined whether ApoBf‐R/E@NPs could interfere with cholesterol homeostasis. ApoBf‐R/E@NP treatment markedly increased the expression of ABCA1, a key regulator of cholesterol efflux (Figure 5H; Figure S6A). Consistently, patient‐derived GBM132 cells displayed elevated ABCA1 expression following ApoBf‐R/E@NP treatment (Figure 5I), suggesting that this lipid regulatory response is preserved in patient‐derived GBM cells. To determine whether ABCA1 elevation resulted from enhanced protein stability, a cycloheximide (CHX) chase assay was performed. ApoBf‐R/E@NPs treatment substantially prolonged the half‐life of ABCA1 compared with control conditions (Figure 5J,K and Figure S6B,C), suggesting that erythrodiol‐mediated intervention stabilizes ABCA1 and is accompanied by depletion of cellular cholesterol pools. This alteration in lipid handling resulted in marked depletion of intracellular lipid reservoirs [22]. Biochemical quantification revealed significant reductions in total cholesterol, free cholesterol, and triglycerides in patient‐derived GBM132 cells (Figure 5L–N), whereas corresponding analyses in LN229 and GL261 cells are shown in Figure S6D–I. These findings were further supported by decreased intracellular lipid droplets observed by fluorescence imaging and quantitative analysis (Figure 5O,P and Figure S6J,K). Together, these results indicate that ApoBf‐R/E@NPs impair both lipid storage capacity and autophagic recycling, thereby restricting the metabolic flexibility required for GBM survival.

To functionally determine whether disruption of autophagic recycling and cholesterol availability jointly contributes to bioenergetic failure, intracellular adenosine triphosphate (ATP) levels were measured under metabolic rescue conditions. ApoBf‐R/E@NP treatment significantly reduced intracellular ATP levels in patient‐derived GBM132 cells (Figure 5Q), with comparable effects observed in LN229 and GL261 cells (Figure S6L,M). Supplementation with exogenous cholesterol (+CHO) or pharmacological inhibition of autophagy initiation with 3‐MA (+3‐MA) partially restored ATP levels. However, simultaneous rescue of both pathways (+CHO+3‐MA) produced a substantially stronger recovery of ATP content in GBM132 cells (Figure 5R) and in established GBM models (Figure S6N,O). These complementation experiments provide mechanistic context for the CI‐defined pharmacological synergy by showing that autophagic recycling and cholesterol availability make separable contributions to ATP maintenance. Regorafenib‐associated restriction of autophagy‐dependent nutrient recycling and erythrodiol‐associated cholesterol depletion impose distinct but convergent metabolic constraints, with ATP depletion emerging as a common downstream bottleneck [53]. Together with the antiangiogenic activity of regorafenib demonstrated above, this combination restricts both extracellular nutrient support and intracellular metabolic salvage while simultaneously limiting cholesterol availability [54, 55]. The resulting bioenergetic deficit therefore reflects complementary disruption of metabolic support systems rather than simply greater exposure to two cytotoxic agents.

### 2.7 Transcriptomic Profiling Links Metabolic Disruption to Survival‐Signaling Suppression and Immune‐Associated Programs

The preceding results suggested that ApoBf‐R/E@NPs impose a coordinated metabolic collapse through autophagic dysfunction, lipid depletion, and ATP loss. We therefore next asked whether these perturbations propagated upward to broader transcriptional programs governing survival signaling, stemness maintenance, and immune regulation. RNA sequencing (RNA‐seq) was performed in LN229 cells to capture the system‐level consequences of concurrent autophagy impairment and ABCA1‐associated lipid efflux (Figure 6A). Volcano plot analysis of the RNA‐seq data revealed extensive transcriptomic reprogramming following ApoBf‐R/E@NPs treatment (Figure 6B). A heatmap of representative differentially expressed genes highlighted coordinated changes in genes linked to cholesterol efflux, autophagy, inflammatory signaling, stemness, and immune evasion (Figure 6C). Among these, *ABCA1*, *SQSTM1*, and *MAP1LC3B* were upregulated together with inflammatory and immune‐interacting genes including *CXCL10*, *CCL5*, and *IFNG*, whereas stemness‐associated and immune‐evasion genes, including *SOX2*, *NANOG*, and *CD274*, were reduced. Kyoto Encyclopedia of Genes and Genomes (KEGG) and Gene Ontology (GO) enrichment analyses further identified significant changes in ATP‐binding cassette transporter, autophagy, PI3K‐AKT, and MAPK pathways, alongside gene sets annotated to cytokine signaling, humoral immune responses, T‐cell differentiation, and related immune processes (Figure 6D,E).

**FIGURE 6:**
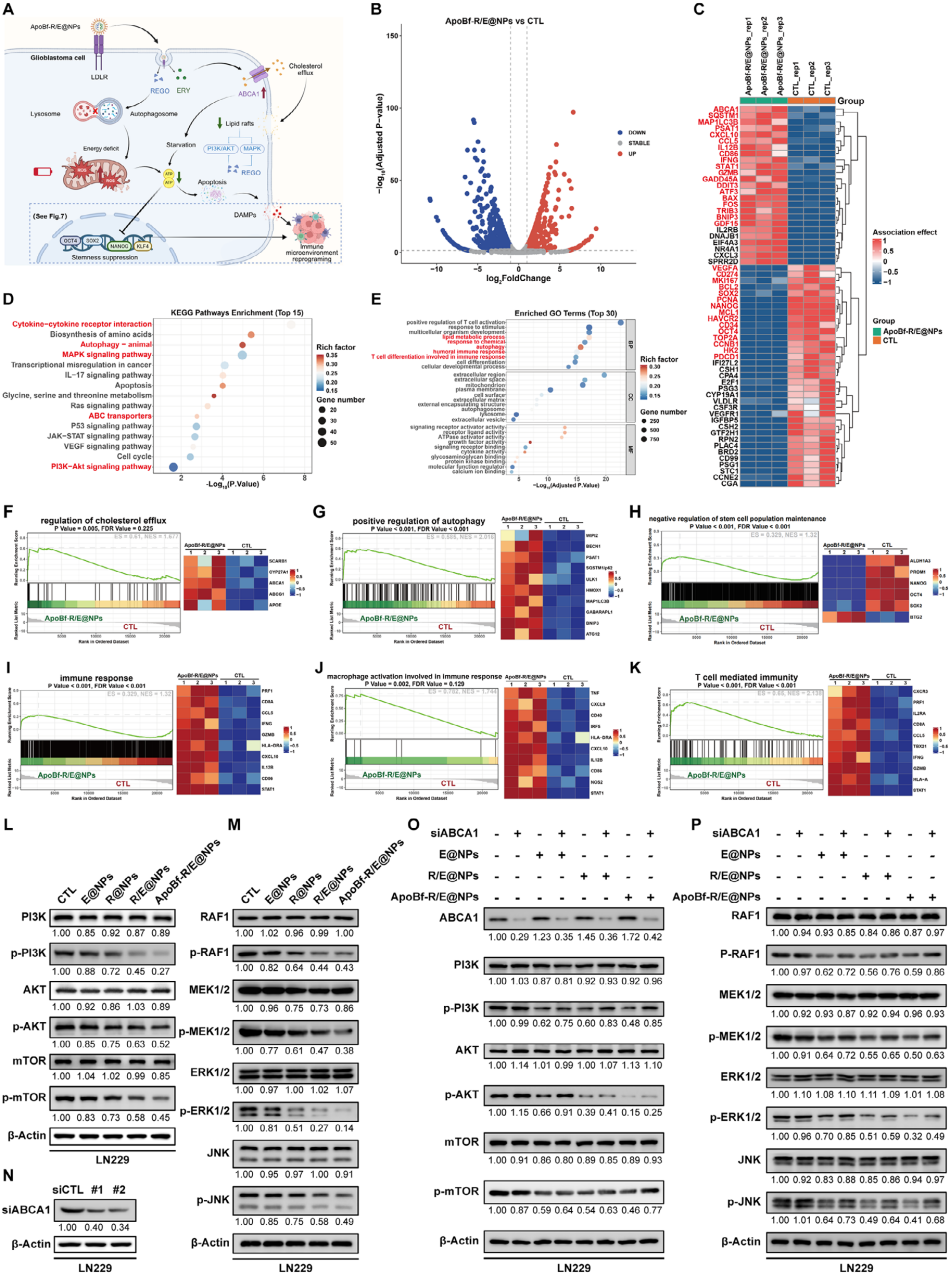
Transcriptomic and biochemical analyses link metabolic disruption to survival‐signaling suppression and immune‐associated programs. (A) Schematic illustration of the proposed metabolic and signaling effects of ApoBf‐R/E@NPs in GBM cells. (B) Volcano plot of differentially expressed genes (DEGs) in ApoBf‐R/E@NP‐treated versus control LN229 cells. (C) Heatmap of representative DEGs associated with cholesterol efflux, autophagy, inflammatory and immune‐related programs, stemness, and immune evasion. (D,E) KEGG pathway enrichment (D) and Gene Ontology (GO) enrichment (E) analyses of DEGs. (F‐K) Gene Set Enrichment Analysis (GSEA) of regulation of cholesterol efflux (F), positive regulation of autophagy (G), negative regulation of stem cell population maintenance (H), immune response (I), macrophage activation involved in immune response (J), and T cell‐mediated immunity (K). Each GSEA panel includes an inset heatmap showing representative genes contributing to the indicated signature. (L, M) Immunoblot analysis of the PI3K/AKT/mTOR (L) and RAF1/MEK/ERK/JNK (M) signaling cascades in LN229 cells following treatment with the indicated formulations. (N) Immunoblot verification of siRNA‐mediated ABCA1 knockdown in LN229 cells. (O, P) Immunoblot analysis of PI3K/AKT/mTOR (O) and RAF1/MEK/ERK/JNK (P) signaling in LN229 cells treated with the indicated formulations in the presence or absence of ABCA1 silencing.

Gene Set Enrichment Analysis (GSEA) further resolved these transcriptional changes. Enrichment of cholesterol‐efflux programs was consistent with the ABCA1‐associated lipid depletion observed above (Figure 6F), whereas enrichment of genes involved in positive regulation of autophagy likely reflects a compensatory transcriptional response occurring in the setting of impaired autophagic flux (Figure 6G). A gene set associated with negative regulation of stem‐cell population maintenance was also enriched following treatment (Figure 6H), consistent with suppression of stemness‐related transcriptional programs. In parallel, ApoBf‐R/E@NP treatment enriched gene sets associated with immune response, macrophage activation, T‐cell‐mediated immunity, and natural killer cell‐mediated cytotoxicity (Figure 6I‐K and Figure S7A). Because these analyses were performed in tumor cells rather than immune‐cell populations, these signatures are best interpreted as an immune‐interacting or immune‐priming transcriptional state rather than direct evidence of immune‐cell activation. Enrichment of apoptotic DNA‐fragmentation genes was also observed (Figure S7B), in agreement with the mitochondrial apoptotic phenotype identified in the preceding experiments.

Transcriptomic changes in PI3K‐AKT and MAPK pathways prompted direct biochemical examination of these survival‐signaling axes. Immunoblotting showed that ApoBf‐R/E@NP treatment markedly reduced phosphorylation within the PI3K/AKT/mTOR and RAF1/MEK/ERK/JNK cascades in LN229 cells (Figure 6L,M), with comparable changes observed in GL261 cells (Figure S7C,D). The suppression was more pronounced with ApoBf‐R/E@NPs than with the corresponding single‐drug or non‐targeted formulations, indicating that the signaling effect accompanied the combined metabolic intervention rather than reflecting regorafenib exposure alone. To determine the contribution of ABCA1‐associated lipid remodeling to this signaling phenotype, ABCA1 was depleted by small interfering RNA (siRNA) in LN229 and GL261 cells (Figure 6N and Figure S7E). ABCA1 silencing attenuated the reduction in PI3K/AKT/mTOR and RAF1/MEK/ERK/JNK phosphorylation induced by ApoBf‐R/E@NPs, producing partial restoration of pathway activity in both cell models (Figure 6O,P and Figure S7F,G). These genetic complementation data support a functional contribution of ABCA1‐dependent lipid remodeling to survival‐signaling suppression. The CI analysis establishes that regorafenib and erythrodiol interact synergistically under the tested 1:1 dosing conditions, whereas the mechanistic experiments clarify the biological basis of this pairing: the two agents restrict distinct but convergent metabolic resources that collectively intensify bioenergetic stress. Regorafenib limits extracellular nutrient support through its antiangiogenic activity and disrupts intracellular nutrient salvage through autophagic dysfunction. In parallel, erythrodiol‐driven ABCA1 stabilization promotes cholesterol efflux and depletes intracellular cholesterol and lipid reserves, thereby limiting lipid availability while weakening cholesterol‐dependent support for PI3K/AKT and MAPK signaling. These complementary perturbations create a nutrient‐ and lipid‐constrained metabolic state that culminates in ATP depletion and attenuation of major survival‐signaling pathways, rather than simply increasing exposure to two cytotoxic agents.

### 2.8 ApoBf‐R/E@NPs Suppress Glioma Stemness and Prime an Immunogenic Microenvironment

Because maintenance of the stem‐like state requires sustained bioenergetic and signaling support [56, 57], we next asked whether the metabolic and signaling stress described above was accompanied by functional suppression of glioma stemness. Three‐dimensional tumorsphere assays showed that ApoBf‐R/E@NPs disrupted spheroid architecture and markedly reduced sphere‐forming capacity in LN229 and GL261 cells (Figure 7A–C and Figure S8A). Flow cytometric analysis likewise revealed a reduced CD133^+^ fraction in LN229 and GL261 cells (Figure 7D and Figure S8B‐D). These changes were interpreted as evidence of suppression of stemness‐associated phenotypes rather than as a quantitative explanation for the overall reduction in tumor‐cell viability. To determine whether ApoBf‐R/E@NPs could directly induce cell death in a stem‐enriched tumor population, patient‐derived GSC178 cultures were examined. Calcein/PI live/dead staining showed a marked increase in PI‐positive red fluorescence following ApoBf‐R/E@NPs treatment, indicating increased cell death (Figure 7E). In parallel, bright‐field tumorsphere analysis demonstrated a pronounced reduction in the number of surviving GSC178 spheres (Figure 7F,G). This phenotypic shift was accompanied by coordinated downregulation of core stemness‐associated proteins, including NOTCH1, OCT4, KLF4, NANOG, and SOX2, in LN229 and GL261 cells (Figure 7H and Figure S8E). Importantly, the same coordinated suppression was reproduced in patient‐derived GBM132 cells and GSC178 cultures (Figure 7I,J). Of note, GSC178 cells also exhibited accumulation of LC3B‐II, SQSTM1/p62, and PSAT1 together with increased ABCA1 abundance following ApoBf‐R/E@NP treatment (Figure 7K), placing the patient‐derived stem‐like compartment within the same autophagy‐lipid remodeling framework established in the preceding GBM models.

**FIGURE 7:**
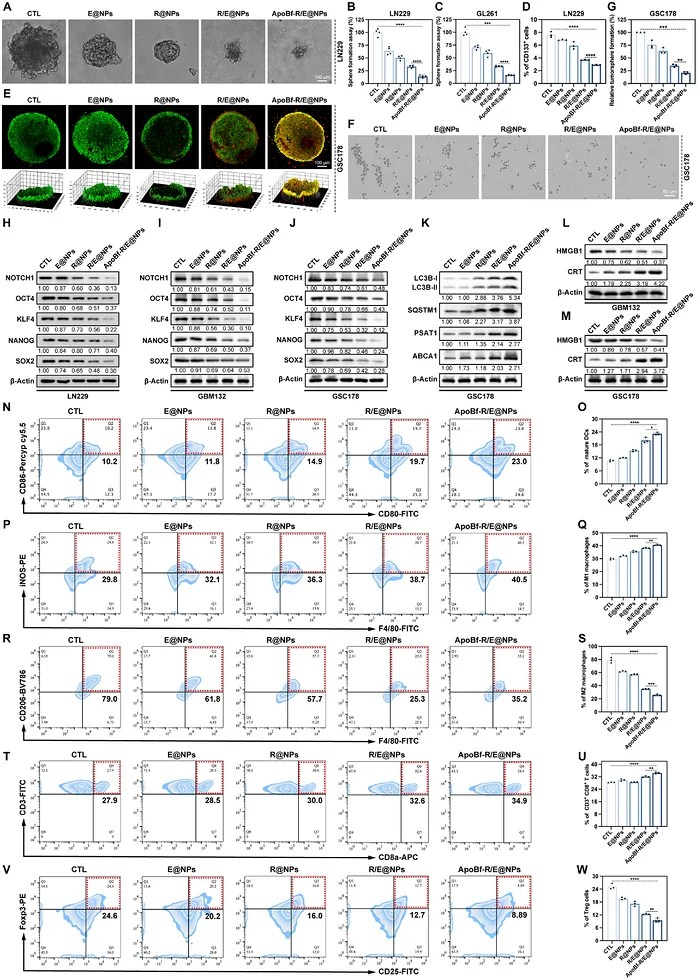
ApoBf‐R/E@NPs suppress glioma stemness and prime an immunogenic microenvironment. (A, B) Representative bright‐field images of LN229 tumorspheres following treatment with the indicated formulations (A) and quantification of relative sphere formation (B). Scale bar, 100 µm. (C) Quantification of relative sphere formation in GL261 cells. (D) Quantification of the CD133^+^ fraction in LN229 cells by flow cytometry. (E) Representative Calcein/PI live/dead fluorescence images of patient‐derived GSC178 tumorspheres, with corresponding three‐dimensional fluorescence‐intensity maps. Calcein identifies viable cells, whereas PI‐positive fluorescence denotes membrane‐compromised dead cells. Scale bar, 100 µm. (F, G) Representative bright‐field images of GSC178 tumorspheres (F) and quantification of relative surviving tumorspheres (G) following treatment. Scale bar, 50 µm. (H‐J) Immunoblot analysis of the stemness‐associated proteins NOTCH1, OCT4, KLF4, NANOG, and SOX2 in LN229 cells (H), patient‐derived GBM132 cells (I), and GSC178 cultures (J). (K) Immunoblot analysis of LC3B, SQSTM1/p62, PSAT1, and ABCA1 in GSC178 cultures. (L, M) Immunoblot analysis of cellular HMGB1 and CRT abundance in whole‐cell lysates from patient‐derived GBM132 cells (L) and GSC178 cultures (M). (N–W) Flow‐cytometric immunophenotyping following co‐culture of pretreated GL261 cells with primary splenocyte and bone marrow‐derived immune fractions isolated from syngeneic mice. Representative plots and corresponding quantification show CD80^+^CD86^+^ mature dendritic cells (N, O), iNOS^+^F4/80^+^ M1 macrophages (P, Q), CD206^+^F4/80^+^ M2 macrophages (R, S), CD3^+^CD8a^+^ T cells (T, U), and CD25^+^Foxp3^+^ regulatory T cells (V, W).

Cells with stem‐like properties are major contributors to the immunosuppressive landscape of GBM, actively maintaining an immunosuppressive barrier through recruitment of regulatory T cells (Tregs) and exclusion of effector T lymphocytes [58, 59]. Suppressing this compartment may therefore weaken tumor‐intrinsic mechanisms that sustain immune exclusion. We next examined whether suppression of stemness‐associated features was accompanied by molecular changes associated with ICD [60]. Immunoblotting showed increased calreticulin (CRT) abundance and reduced cellular high‐mobility group box 1 (HMGB1) levels in patient‐derived GBM132 cells and GSC178 cultures (Figure 7L,M), with concordant changes in LN229 and GL261 cells (Figure S8F,G). These alterations are consistent with an ICD‐associated tumor‐cell stress response and provide a potential interface between treatment‐induced tumor damage and innate immune recognition [61]. To determine whether stemness suppression and the accompanying ICD‐associated tumor‐cell changes translated into functional immune remodeling, GL261 cells were pretreated with the indicated formulations and subsequently co‐cultured with primary splenocyte and bone marrow‐derived immune fractions isolated from syngeneic mice. Flow cytometric profiling demonstrated a marked increase in CD80^+^CD86^+^ mature dendritic cells (DCs) following exposure to ApoBf‐R/E@NP‐treated GL261 cells (Figure 7N,O). DC maturation was accompanied by remodeling of the macrophage compartment, with enrichment of iNOS^+^F4/80^+^ M1 macrophages and a reciprocal reduction in CD206^+^F4/80^+^ M2 macrophages (Figure 7P‐S). The adaptive immune compartment shifted in parallel, with increased frequencies of CD3^+^CD8a^+^ T cells (Figure 7T,U) and CD3^+^CD4^+^ T cells (Figure S8H,I), together with a marked reduction in CD25^+^Foxp3^+^ Tregs (Figure 7V,W).

Because ApoB‐frags constitute the LDLR‐recognition interface of the nanoplatform, we further examined whether the unloaded ApoBf‐functionalized carrier itself contributed to the biological effects observed above. TEM and DLS confirmed the formation of unloaded ApoBf@NPs with a nanoscale particulate morphology and a Z‐average hydrodynamic diameter of 157.3 nm (Figure S9A,B). ApoBf@NPs alone had little effect on LN229 cell viability across the tested carrier‐equivalent concentrations and did not appreciably alter LC3B‐II, SQSTM1/p62, PSAT1, ABCA1, or the stemness‐associated proteins NOTCH1, OCT4, KLF4, NANOG, and SOX2 (Figure S9C–F). In contrast, these metabolic and stemness‐associated changes were evident in the drug‐loaded groups. Consistent with these tumor‐cell‐intrinsic findings, unloaded ApoBf@NPs did not significantly alter DC maturation, M1/M2 macrophage polarization, CD8^+^ or CD4^+^ T‐cell frequencies, or the Treg population in the GL261‐immune‐cell co‐culture system, whereas clear remodeling of these immune populations was observed in the drug‐loaded groups (Figure S9G–R). These results show that unloaded ApoBf@NPs do not independently account for the metabolic, stemness‐associated, or immunological changes observed with ApoBf‐R/E@NPs under the tested conditions. The additional effects associated with ApoBf functionalization are therefore more consistent with enhanced delivery of the encapsulated payloads than with an intrinsic biological activity of the unloaded carrier. Together, these in vitro and ex vivo findings connect disruption of autophagy‐lipid homeostasis with suppression of the stem‐like GBM state and coordinated remodeling of innate and adaptive immune compartments, thereby providing a mechanistic basis for the antitumor immune responses examined in the subsequent in vivo models.

### 2.9 ApoBf‐R/E@NPs Suppress Orthotopic GBM Progression and Remodel the In Situ Microenvironment

Having established that ApoBf‐R/E@NPs suppress stem‐like GBM features and promote an immunogenic tumor‐cell state in vitro, we next examined whether these effects translated into therapeutic activity in an immunocompetent orthotopic setting. GL261‐Luc cells were stereotactically implanted into the striatum of C57BL/6 mice to establish intracranial tumors (Figure 8A). Because effective GBM therapy requires both BBB traversal and accumulation within the tumor‐bearing brain [62], we first examined the spatiotemporal distribution of the formulation in vivo. Compared with untargeted R/E@NPs, ApoBf‐R/E@NPs produced a stronger cranial fluorescence signal (Figure 8B), which peaked at approximately 8 h after administration and remained detectable for up to 48 h (Figure 8C). Ex vivo imaging further showed prominent brain‐associated fluorescence together with hepatic and renal distribution (Figure 8D,E), supporting enhanced intracranial exposure of the ApoBf‐functionalized formulation. We then assessed whether this delivery advantage was accompanied by improved tumor control. Longitudinal bioluminescence imaging revealed progressive intracranial tumor expansion in control mice, whereas ApoBf‐R/E@NP treatment produced sustained suppression of tumor‐associated bioluminescence relative to the other treatment groups (Figure 8F,G). This effect was accompanied by a significant extension of overall survival (Figure 8H), while body weight remained stable throughout the treatment period (Figure 8I).

**FIGURE 8:**
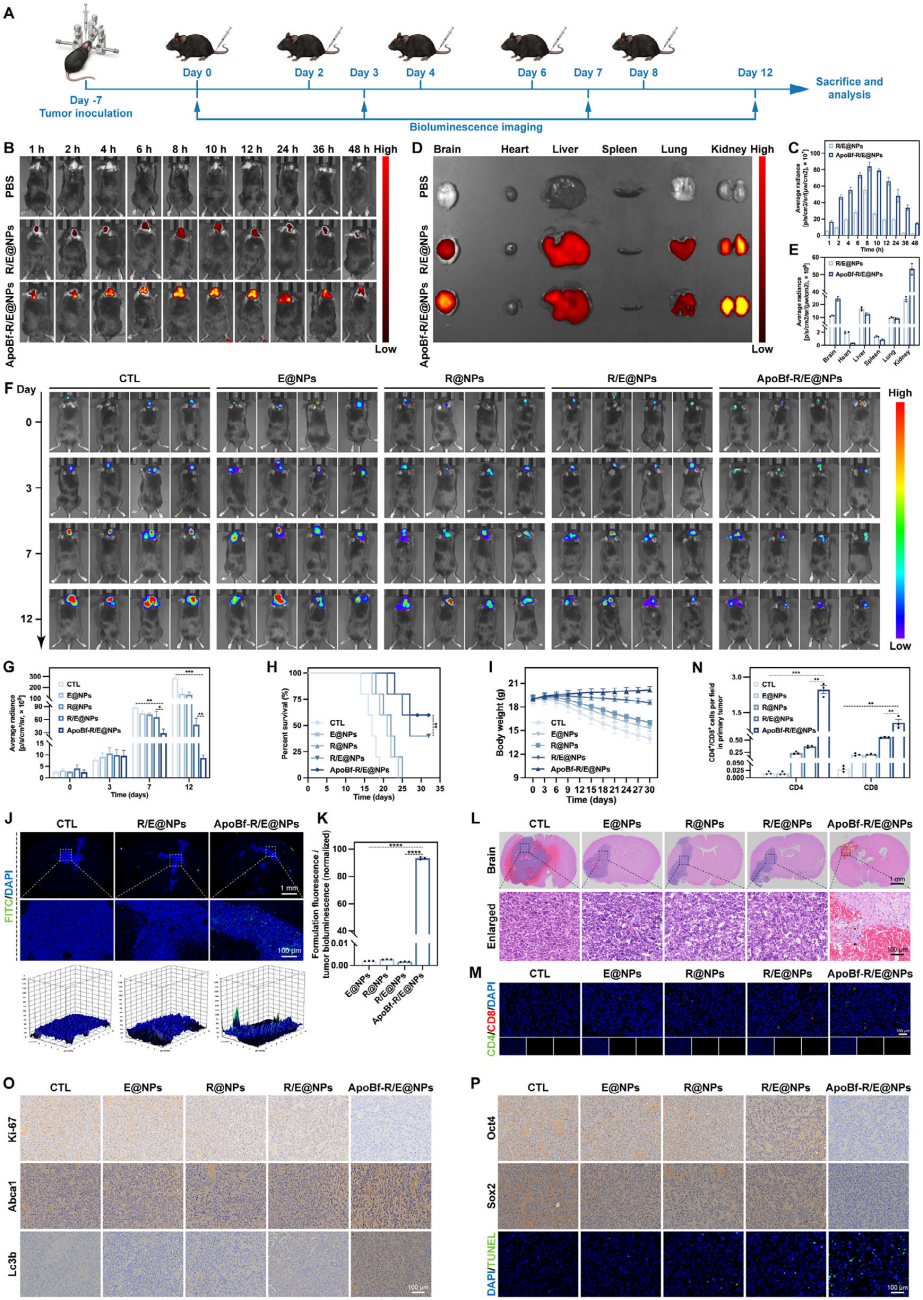
ApoBf‐R/E@NPs preferentially accumulate in intracranial GBM, suppress orthotopic tumor progression, and remodel the local tumor microenvironment. (A) Schematic illustration of the establishment of the immunocompetent orthotopic GL261‐Luc glioma model in C57BL/6 mice and the treatment and imaging schedule. (B,C) Longitudinal in vivo fluorescence imaging of mice receiving PBS, R/E@NPs, or ApoBf‐R/E@NPs (B) and corresponding quantification of cranial formulation‐associated fluorescence over time (C). (D, E) Ex vivo fluorescence imaging of the brain and major organs (D) and corresponding quantitative analysis of tissue‐associated fluorescence (E). (F, G) Longitudinal bioluminescence imaging of intracranial GL261‐Luc tumor progression in the indicated treatment groups (F) and quantitative analysis of tumor‐associated bioluminescence (G). (H) Kaplan‐Meier survival analysis of mice receiving the indicated treatments. (I) Longitudinal monitoring of body weight during the treatment period. (J) Confocal fluorescence mapping of coronal brain sections showing the spatial distribution of DSPE‐PEG‐FITC‐labeled formulations, together with enlarged views of the indicated regions and corresponding three‐dimensional fluorescence‐intensity maps. Scale bars, 1 mm and 100 µm. (K) Formulation fluorescence normalized to tumor bioluminescence at 12 h after administration. (L) Representative hematoxylin and eosin (H&E)‐stained coronal brain sections and enlarged views of the intracranial tumor regions. Scale bars, 1 mm and 50 µm. (M, N) Representative immunofluorescence images of intratumoral CD4^+^ and CD8^+^ T lymphocytes (M) and corresponding quantitative analysis (N). Scale bar, 100 µm. (O) Representative IHC staining for Ki‐67, Abca1, and Lc3b in orthotopic tumor tissues. Scale bar, 100 µm. (P) Representative IHC staining for Oct4 and Sox2 and TUNEL staining of orthotopic tumor sections. Scale bar, 100 µm.

To determine more directly whether the cerebral fluorescence was associated with the intracranial tumor rather than diffusely distributed throughout the brain, fluorescence mapping of coronal brain sections was performed. ApoBf‐R/E@NPs showed pronounced FITC‐associated fluorescence within the tumor‐bearing region, with comparatively limited signal in the surrounding brain parenchyma (Figure 8J). This spatial distribution was further examined by dual‐modal imaging at 12 h after administration, in which formulation‐associated fluorescence was compared with the bioluminescence signal generated by GL261‐Luc tumors. The fluorescence hotspot of ApoBf‐R/E@NPs closely corresponded to the bioluminescence imaging (BLI)‐defined tumor region (Figure S10A), and the normalized formulation fluorescence‐to‐tumor bioluminescence ratio was markedly higher in the ApoBf‐R/E@NP group (Figure 8K). Together with the brain‐section imaging, these data provide quantitative support for preferential accumulation of the targeted formulation within intracranial tumors. Because DSPE‐PEG‐FITC served as a lipid‐associated tracer, the fluorescence signal was interpreted as a readout of the labeled formulation rather than as an independent measure of intact nanoparticle fate. Hematoxylin and eosin (H&E) staining further showed a substantial reduction in intracranial tumor burden following ApoBf‐R/E@NP treatment (Figure 8L). Immunofluorescence analysis revealed increased infiltration of CD8^+^ cytotoxic and CD4^+^ helper T lymphocytes within the tumor tissue (Figure 8M,N), consistent with the immune remodeling observed in the preceding ex vivo experiments. We next examined whether the metabolic and phenotypic effects identified in vitro were retained within the orthotopic tumor. ApoBf‐R/E@NP treatment reduced Ki‐67 staining and increased Abca1 and Lc3b expression (Figure 8O and Figure S10B‐D), consistent with suppression of proliferation and engagement of the lipid‐ and autophagy‐associated pathways targeted by the formulation. In parallel, Oct4 and Sox2 expression was reduced, whereas terminal deoxynucleotidyl transferase dUTP nick‐end labeling (TUNEL) positivity was markedly increased (Figure 8P and Figure S10E‐G). These tissue‐level findings therefore connect intracranial delivery of ApoBf‐R/E@NPs with suppression of stemness‐associated features, local immune‐cell infiltration, and tumor‐cell death in vivo.

Given the hepatic and renal fluorescence detected in the biodistribution analysis, together with the physiological involvement of LDLR‐related lipid transport pathways, we further evaluated systemic safety under the therapeutic regimen. Serum biochemical analyses showed no significant treatment‐associated alterations across multiple indices of organ function and systemic metabolic homeostasis, including hepatic parameters (ALB, ALP, ALT, AST, DBil, TBil, and TP), renal parameters (Crea, UA, and UREA), myocardial and tissue‐injury markers (CKMB and LDH), pancreatic enzymes (AMY and LIP), glucose and lipid metabolic indices (GLU, CHOL, HDL, LDL, and TG), and the inflammatory marker CRP (Figure S11A–T). Consistent with these biochemical findings, histopathological examination of the brain, heart, and liver (Figure S11U), as well as the spleen, lungs, and kidneys (Figure S11V), revealed no overt treatment‐associated morphological abnormalities. Collectively, these results demonstrate that ApoBf‐R/E@NPs achieve preferential intracranial tumor‐associated delivery and orthotopic tumor control while reproducing the metabolic, stemness‐related, and immune effects identified in vitro, with a favorable systemic safety profile under the tested conditions.

### 2.10 ApoBf‐R/E@NPs Mobilize Systemic Antitumor Immunity to Suppress Distal, Treatment‐Naive Lesions

Following the local immune remodeling observed in the orthotopic GBM model, we next asked whether ApoBf‐R/E@NP treatment could generate antitumor effects beyond the treated lesion. To address this question, we established a bilateral subcutaneous GL261 model in immunocompetent C57BL/6 mice. By inoculating tumors on both flanks and restricting intratumoral treatment to the right‐flank primary lesion, we were able to monitor the contralateral untreated tumor as a readout of systemic antitumor activity (Figure 9A). Macroscopic and quantitative tracking revealed that localized intervention effectively suppressed the treated primary tumors, as evidenced by substantial reductions in terminal weight, absolute volume, and longitudinal growth kinetics (Figure 9B‐E). Consistent with these findings, histological examination of the primary lesions showed treatment‐associated structural disruption and reduced Ki‐67 staining (Figure S12Q,R). Notably, this focal treatment also restrained the growth of the distant, non‐injected lesions. Distal efficacy was manifested by smaller explants at necropsy (Figure 9F), together with significant reductions in tumor weight (Figure 9G), ex vivo volume (Figure 9H), and longitudinal growth kinetics (Figure 9I). Because the contralateral tumors received no direct treatment, their concomitant suppression, together with the immune changes described below, was consistent with the induction of a systemic antitumor response.

**FIGURE 9:**
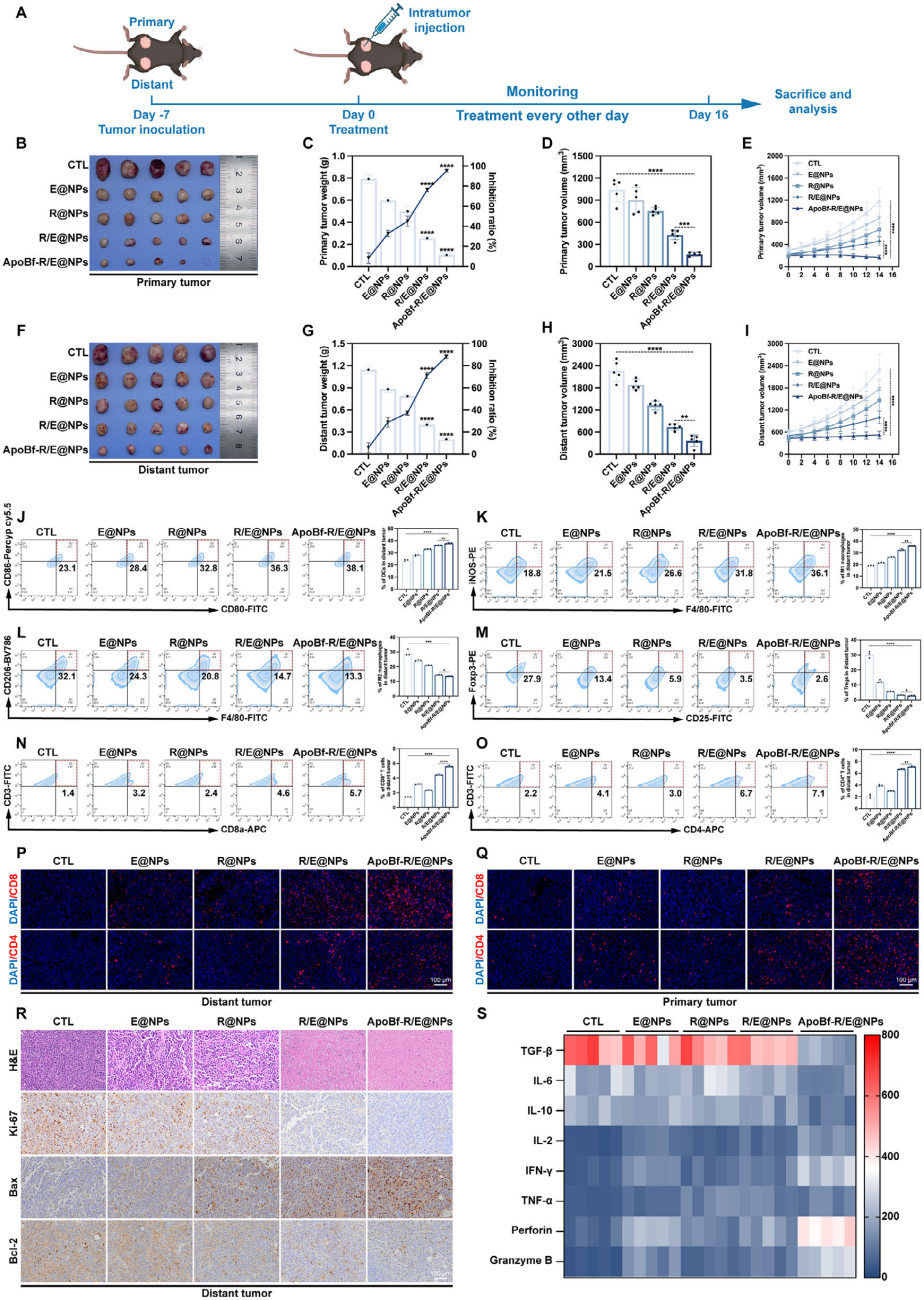
Local ApoBf‐R/E@NPs treatment suppresses untreated distal tumors and mobilizes systemic antitumor immunity in a bilateral GL261 model. (A) Schematic representation of the immunocompetent bilateral GL261 GBM model, in which the right‐flank primary tumor received localized intratumoral treatment while the contralateral distal tumor remained untreated. (B–E) Parallel assessment of the primary (treated) tumors, presenting macroscopic images of the excised tumors (B), terminal weights and corresponding tumor‐inhibition ratios (C), ex vivo volumes (D), and longitudinal tumor growth curves (E). (F–I) Macroscopic and quantitative evaluation of the distal (untreated) tumors, including representative photographs of excised tumors (F), terminal tumor weights and corresponding tumor‐inhibition ratios (G), ex vivo volumes (H), and longitudinal tumor growth kinetics (I). (J–O) Flow‐cytometric immunophenotyping of the distal tumor microenvironment, showing CD80^+^CD86^+^ mature DCs (J), iNOS^+^F4/80^+^ M1 macrophages (K), CD206^+^F4/80^+^ M2 macrophages (L), CD25^+^Foxp3^+^ Tregs (M), CD3^+^CD8a^+^ T cells (N), and CD3^+^CD4^+^ T cells (O), together with the corresponding quantification. (P, Q) Representative immunofluorescence images of CD8^+^ and CD4^+^ T‐cell infiltration in distal (P) and primary (Q) tumors. Scale bars, 100 µm. (R) Representative H&E and IHC staining for Ki‐67, Bax, and Bcl‐2 in untreated distal tumors. Scale bar, 100 µm. (S) Heatmap of serum cytokine concentrations measured by enzyme‐linked immunosorbent assay (ELISA), including TGF‐β, IL‐6, IL‐10, IL‐2, IFN‐γ, TNF‐α, perforin, and granzyme B, concentration (pg/mL).

To identify the immune architecture underlying this distal response, we performed comprehensive immunological profiling across the distal and proximal tumor microenvironments as well as peripheral myeloid and lymphoid compartments. Flow cytometric analysis of the distal tumors revealed increased frequencies of mature DCs and pro‐inflammatory M1 macrophages (Figure 9J,K), accompanied by reductions in immunosuppressive M2 macrophages and Tregs (Figure 9L,M). CD8^+^ cytotoxic and CD4^+^ helper T lymphocytes were likewise increased within the untreated distal tumors (Figure 9N,O). Similar immune changes were observed in the treated primary tumors (Figure S12A‐F). In peripheral immune compartments, myeloid populations were assessed in bone marrow‐derived cells (Figure S12G‐I), whereas Tregs and CD8^+^ and CD4^+^ T‐cell populations were profiled in splenocytes (Figure S12J‐L), revealing changes concordant with those observed within the tumors. Complementary immunofluorescence mapping further demonstrated increased intratumoral densities of CD8^+^ and CD4^+^ T cells in both distal and primary lesions (Figure 9P,Q and Figure S12M‐P).

Consistent with the inhibition of distal tumor growth, histological and immunohistochemical analyses revealed marked structural disruption, reduced Ki‐67 staining, increased Bax expression, and decreased Bcl‐2 expression in the untreated tumors (Figure 9R and Figure S12S‐U). Serum cytokine analysis further supported systemic immune remodeling. ApoBf‐R/E@NPs treatment was associated with increased circulating interleukin‐2 (IL‐2), interferon‐γ (IFN‐γ), tumor necrosis factor‐α (TNF‐α), perforin, and granzyme B, together with reduced transforming growth factor‐β (TGF‐β), interleukin‐6 (IL‐6), and interleukin‐10 (IL‐10, Figure 9S). The systemic decline of TGF‐β and IL‐10, two soluble immunoregulatory factors closely associated with T cell dysfunction and immune exclusion [63, 64], is consistent with attenuation of systemic immunoregulatory signaling. Throughout the therapeutic window, the body weight of the subjects remained stable across all cohorts (Figure S12V), with no evident treatment‐associated weight loss. Taken together, these findings show that local ApoBf‐R/E@NP treatment suppresses untreated distal tumor growth in association with coordinated remodeling of innate and adaptive immune compartments and a systemic shift toward an antitumor cytokine profile.

### 2.11 ApoBf‐R/E@NPs Delay Postsurgical GBM Recurrence and Are Associated With Memory T‐Cell Expansion

Having established that local ApoBf‐R/E@NP treatment can generate systemic antitumor responses against untreated distal lesions, we next asked whether this immune remodeling could be maintained in a postsurgical setting and contribute to the control of residual disease. To examine whether ApoBf‐R/E@NPs could control tumor regrowth from residual disease after surgery, we established a subcutaneous GL261‐Luc subtotal resection model in immunocompetent C57BL/6 mice (Figure 10A). GL261‐Luc tumors were allowed to reach 500–600 mm^3^ before subtotal resection, after which residual tumor burden was assessed by bioluminescence imaging. Representative gross images of the postoperative tumor beds are shown in Figure S13A. Post‐resection BLI confirmed the presence of residual GL261‐Luc signals before postoperative treatment (Figure 10B), providing an imaging baseline from which subsequent tumor regrowth could be evaluated. Following treatment, recurrent tumors showed markedly different bioluminescence burdens across the experimental groups, with the lowest signals observed in mice receiving ApoBf‐R/E@NPs (Figure 10C). Recurrent lesions collected at endpoint were likewise smaller following targeted treatment (Figure 10D). Quantitative comparison of tumor bioluminescence between the post‐resection baseline and the recurrence stage showed a substantial increase in tumor bioluminescence in the control and single‐agent groups, whereas this increase was strongly attenuated by R/E@NPs and ApoBf‐R/E@NPs (Figure 10E). Consistent with the imaging data, ApoBf‐R/E@NPs treatment resulted in the lowest recurrent tumor weight and the highest corresponding inhibition ratio (Figure 10F), together with marked suppression of longitudinal tumor regrowth (Figure 10G) and reduced recurrent tumor volume at endpoint (Figure 10H). Survival analysis further showed prolonged overall survival following ApoBf‐R/E@NPs treatment (Figure S13B). Histopathological assessment of recurrent tumors revealed reduced Ki‐67 staining together with increased intratumoral CD8^+^ and CD4^+^ T‐cell infiltration (Figure S13C–F). Body weight remained stable during postoperative treatment (Figure S13G), with no evident treatment‐associated weight loss.

**FIGURE 10:**
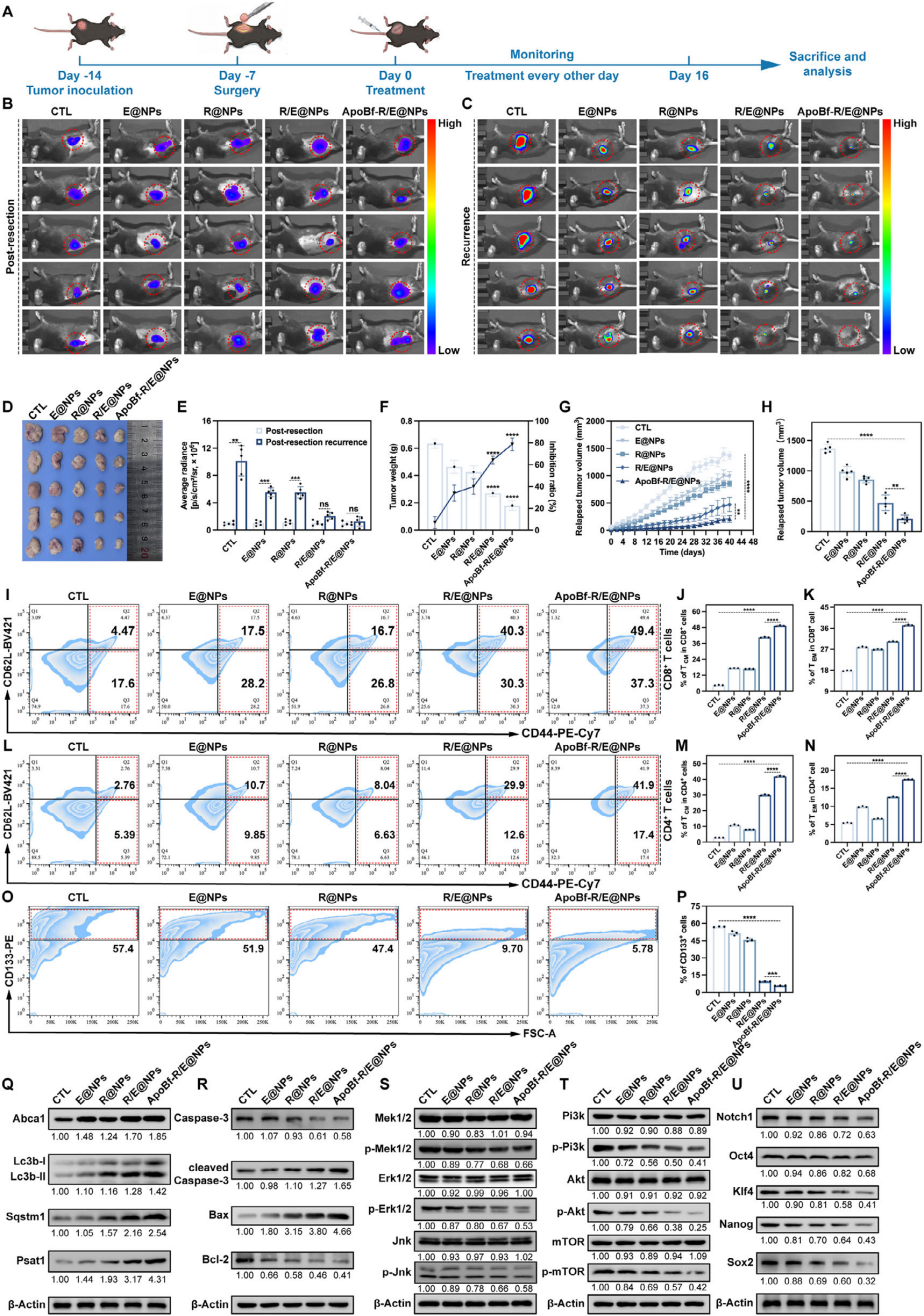
ApoBf‐R/E@NPs delay postsurgical GBM recurrence and are associated with memory T‐cell expansion and suppression of the stem‐like compartment. (A) Schematic illustration of the GL261‐Luc subtotal‐resection model and postoperative treatment schedule in immunocompetent C57BL/6 mice. (B) Bioluminescence imaging of residual GL261‐Luc tumors following subtotal resection and before postoperative treatment. (C) Representative bioluminescence imaging of recurrent tumors following the indicated postoperative treatments. (D) Representative photographs of recurrent tumors collected at endpoint. (E) Quantitative comparison of tumor bioluminescence at the post‐resection baseline and recurrence stage in each treatment group. (F) Recurrent tumor weights and corresponding tumor‐inhibition ratios at endpoint. (G) Longitudinal growth curves of recurrent tumors during postoperative treatment. (H) Recurrent tumor volumes at endpoint. (I‐N) Flow cytometric profiling of memory T‐cell subsets within recurrent tumors. Representative CD44/CD62L profiles and corresponding quantification show central memory (T_CM_, CD44^+^/CD62L^+^) and effector memory (T_EM_, CD44^+^/CD62L^−^) populations within the CD8^+^ (I‐K) and CD4^+^ (L‐N) T‐cell compartments. (O,P) Flow‐cytometric analysis of CD133^+^ cells in recurrent tumors (O) and corresponding quantification (P). (Q–U) Immunoblot analyses of recurrent tumor tissues showing Abca1, Lc3b‐I/II, Sqstm1, and Psat1 (Q); Caspase‐3/cleaved Caspase‐3, Bax, and Bcl‐2 (R); Mek1/2, Erk1/2, Jnk and their phosphorylated forms (S); Pi3k, Akt, mTOR and their phosphorylated forms (T); and the stemness‐associated proteins Notch1, Oct4, Klf4, Nanog, and Sox2 (U).

Given the systemic immune remodeling observed in the bilateral model, we next examined whether immune activation persisted within recurrent tumors and across peripheral immune compartments during postoperative tumor control. Immunophenotyping of the recurrent tumor microenvironment showed increased frequencies of mature DCs and M1 macrophages, accompanied by reductions in M2 macrophages and Tregs and increased CD8^+^ and CD4^+^ T‐cell populations (Figure S13H–M). Similar myeloid changes were detected in bone marrow‐derived cells (Figure S13N–P), whereas splenic immune profiling showed reduced Tregs together with increased CD8^+^ and CD4^+^ T‐cell populations (Figure S13Q–S). Flow cytometric analysis further revealed coordinated expansion of both central memory T cells (T_CM_, CD44^+^/CD62L^+^) and effector memory T cells (T_EM_, CD44^+^/CD62L^−^) within the CD8^+^ and CD4^+^ compartments (Figure 10I–N). The enrichment of these memory‐associated T‐cell populations within recurrent tumors, together with parallel immune remodeling in the spleen and bone marrow, supports the persistence of a broader immune‐active state during postoperative tumor control [65, 66].

We additionally examined whether the molecular effects associated with treatment were retained in recurrent tumors. Flow cytometric analysis showed a reduced CD133^+^ fraction following ApoBf‐R/E@NPs treatment (Figure 10O,P). Immunoblotting of recurrent tumor tissues further showed increased Abca1 together with accumulation of Lc3b‐II, Sqstm1, and Psat1 (Figure 10Q), accompanied by increased cleaved Caspase‐3 and Bax, reduced Bcl‐2 (Figure 10R), suppression of Mek/Erk/Jnk and PI3K/AKT/mTOR signaling (Figure 10S,T), and decreased expression of Notch1, Oct4, Klf4, Nanog, and Sox2 (Figure 10U). These findings indicate that the metabolic, apoptotic, signaling, and stemness‐associated changes identified in the preceding models were also retained within recurrent tumors. Collectively, the postsurgical model demonstrates that ApoBf‐R/E@NPs delay regrowth from residual disease while sustaining broad antitumor immune activation and memory‐associated T‐cell responses, with the expected pharmacodynamic changes retained in recurrent tumors.

Several limitations should be considered when interpreting the translational scope of the present study. First, although LDLR was retained across multiple GBM contexts, its abundance varied among molecular subtypes, malignant cell states, and experimental models, indicating that the magnitude of ApoBf‐mediated delivery is unlikely to be uniform across tumors. Second, BBB transport and in vivo therapeutic efficacy were modeled predominantly in murine systems, including bEnd.3‐based transendothelial transport and syngeneic GL261 tumors. Although these models enable mechanistic interrogation in an immunocompetent setting, they do not fully reproduce the biological complexity of the human BBB or the genetic, vascular, stromal, and immune heterogeneity of patient GBM, which should be considered when extrapolating the observed delivery and therapeutic effects. Third, the favorable systemic tolerance observed under the present treatment schedules defines an encouraging preclinical safety window but does not by itself establish the tolerability of prolonged systemic administration in a clinical setting. Finally, because experimentally selected drug‐resistant GBM models were not included, the present findings do not establish reversal of acquired drug resistance. Within these boundaries, the principal contribution of this work lies in integrating BBB‐oriented nanodelivery with complementary disruption of autophagy‐associated nutrient salvage and cholesterol‐dependent metabolic support, thereby achieving broad GBM control while suppressing stemness‐associated programs and promoting antitumor immune remodeling. These findings provide a formulation framework for translating mechanistically rational drug combinations into brain‐tumor‐directed nanomedicines.

## 3 Conclusion

In summary, this study establishes ApoBf‐R/E@NPs as an LDLR‐oriented biomimetic nanoplatform that couples BBB‐oriented brain‐tumor delivery with complementary metabolic intervention. Regorafenib and erythrodiol exhibit a synergistic pharmacological interaction and, when co‐delivered, impose convergent metabolic pressure by restricting autophagy‐associated nutrient salvage and cholesterol‐dependent metabolic support, while the antiangiogenic activity of regorafenib further limits extracellular nutrient supply. These complementary perturbations converge on ATP depletion, mitochondrial dysfunction, suppression of PI3K/AKT and MAPK signaling, and apoptotic cell death, thereby broadly impairing GBM growth while weakening stemness‐associated programs and reducing the fitness of stem‐like tumor cells. Through LDLR engagement, the ApoB‐frag interface enhances transendothelial transport and preferential tumor‐associated delivery, enabling the co‐loaded metabolic intervention to more effectively access intracranial GBM. At the tumor level, this integrated delivery‐and‐treatment strategy suppresses orthotopic GBM progression, restrains untreated distal tumor growth, and delays regrowth from residual disease after surgery. These therapeutic effects are accompanied by an ICD‐associated tumor‐cell state, remodeling of local and systemic antitumor immunity, and expansion of memory‐associated T‐cell populations. Collectively, our findings establish a therapeutic framework in which BBB‐oriented nanodelivery is mechanistically matched to complementary metabolic vulnerabilities to achieve broad GBM control while retaining activity against the stem‐like compartment and promoting antitumor immunity.

## 4 Experimental Section

### 4.1 Materials and Reagents

Regorafenib (HY‐10331), erythrodiol (HY‐N2419), and cycloheximide (HY‐12320) were obtained from MedChemExpress. DSPE‐PEG (D163634) and DSPE‐PEG‐FITC (D163610) were supplied by Aladdin Biotech. Recombinant human apolipoprotein B fragment (RPU40344, BIOMATIK), containing a defined LDLR‐binding sequence within His3365‐Glu3548, was used as an apolipoprotein‐derived interfacial ligand to provide LDLR recognition while reducing the steric burden associated with full‐length ApoB during nanoparticle assembly [67]. DQ‐BSA probe (D‐12051) was acquired from Thermo Fisher Scientific, and D‐Luciferin Potassium Salt (40902ES09) from Yeasen Biotechnology. A comprehensive suite of cellular assays and probes, including CCK‐8 (C0038), EdU (C0085), LDH (C0016), cell cycle (C1052), DCFH‐DA (S1105), JC‐1 (C2006), cholesterol (S0211), triglyceride (S0219), lipid droplets (C2053), and ATP (S0027) detection kits, was purchased from Beyotime Biotechnology. The Annexin V‐FITC/PI apoptosis detection kit (556547) was obtained from BD Biosciences. Cytokine quantification was performed using ELISA kits for IL‐2, IL‐6, IL‐10, IFN‐γ, Perforin, TGF‐β, and TNF‐α (ABclonal Technology Co., Ltd.), alongside a Granzyme B kit (EK1115, Boster Biological Technology).

Antibodies targeting cell cycle, apoptosis, and stemness networks included: CDK‐2 (A27938), PARP1 (A0942), β‐Actin (AC026), β‐Tubulin (A12289), NOTCH1 (R380355), and Calreticulin (A20986) from ABclonal; cleaved PARP‐1 (CY5035), KLF4 (CY6648), and SOX2 (CY5268) from ABWAYS; Caspase‐3 (9662) and cleaved Caspase‐3 (Asp175, 9661) from Cell Signaling Technology; BAX (T40051) and BCL‐2 (T40056F) from Abmart; and ABCA1 (ab18180), OCT4 (ab200834), and NANOG (ab317506) from Abcam. Antibodies for autophagic and metabolic assessment included LC3B (NB100‐233, Novus Biologicals), SQSTM1 (R25788), and LAMP1 (516894) from ZENBIOSCIENCE, and PSAT1 (10501‐1‐AP) from Proteintech. Kinase signaling cascades were probed using antibodies against RAF1/p‐RAF1, MEK1/2/p‐MEK1/2, JNK/p‐JNK, PI3K/p‐PI3K, AKT/p‐AKT, mTOR/p‐mTOR, ERK1/2/p‐ERK1/2, and HMGB1 (all sourced from ZENBIOSCIENCE). Fluorochrome‐conjugated antibodies for flow cytometric immunophenotyping (anti‐CD133, CD11b, CD11c, CD80, CD86, F4/80, iNOS, CD206, CD4, CD25, Foxp3, CD3, CD8a, CD44, and CD62L) were all purchased from BioLegend and utilized according to the manufacturer's titration protocols.

### 4.2 Supramolecular Engineering and Interfacial Optimization of ApoBf‐R/E@NPs

ApoBf‐R/E@NPs were formulated via a thin‐film hydration method followed by amphipathic interfacial anchoring. Briefly, regorafenib (1.11 mg, 2.30 µmol) and erythrodiol (1.02 mg, 2.30 µmol), strictly maintaining an optimized equimolar ratio, alongside the amphiphilic matrix DSPE‐PEG (12.0 mg), were co‐dissolved in 2.0 mL of a chloroform/methanol solvent mixture (2:1, v/v). The organic phase was subsequently evaporated under reduced pressure at 45°C using a rotary evaporator and subjected to overnight vacuum desiccation to rigorously remove residual solvents. This process effectively prevented macroscopic crystallization of the hydrophobic payloads, yielding a uniform, molecularly dispersed drug‐loaded lipid film. To drive nanoscale co‐assembly, the dried film was hydrated with 2.0 mL of deionized water and vigorously vortexed to form a crude suspension. The suspension was then processed using a probe sonicator (100 W; 3 s ON / 3 s OFF cycle) in an ice bath for 5 min. To remove undispersed macroscopic drug crystals and potential titanium debris shed from the sonicator probe, the dispersion was centrifuged at 10 000 × g for 10 min. The supernatant containing pristine R/E@NPs was collected for subsequent biointerfacial functionalization. For fluorescence tracking, DSPE‐PEG‐FITC was incorporated into the lipid matrix during nanoparticle assembly at 2.0 mol% of total DSPE‐PEG.

To determine the optimal protein coating density for targeting fidelity, a titration‐based screening was conducted. ApoB‐frag (His3365‐Glu3548) was incubated with preformed R/E@NPs at varying concentration gradients (0.25 to 8.0 µg per mg of lipid) in 10 mM HEPES buffer (pH 7.4) at 4 °C for 12 h under gentle rotation (60 rpm). This post‐insertion strategy was designed to favor amphipathic interfacial association with the lipid‐PEG assembly while preserving aqueous exposure of the LDLR‐recognition region, rather than burying the ligand within the hydrophobic core. The structural evolution, colloidal stability, and surface charge of the resulting assemblies were monitored via DLS and electrophoretic mobility measurements. Based on the near‐plateau in surface potential at 2.0 µg/mg and the progressive increase in particle size at higher ApoB‐frag inputs, 2.0 µg ApoB‐frags per mg lipid was selected as the standardized configuration for all subsequent experiments. The final ApoBf‐R/E@NPs were purified using a Sephadex G‐50 SEC column eluted with 1× PBS (pH 7.4) to isolate the assembled nanoplatform from unassociated ApoB‐frags and unencapsulated drugs. Protein retention and structural integrity were continuously verified by tracking the ultraviolet absorbance at 280 nm (A280) and the lipidic FITC signal.

For carrier‐control experiments, unloaded ApoBf‐functionalized nanoparticles (ApoBf@NPs) were prepared in parallel using the same procedure but without regorafenib or erythrodiol. ApoBf@NPs were characterized by TEM and DLS using the same procedures as the drug‐loaded formulation and were applied at carrier‐equivalent concentrations matched to the corresponding ApoBf‐R/E@NP groups.

### 4.3 Physicochemical Characterization and Drug Loading Quantification

The hydrodynamic diameter and zeta potential were determined using a Zetasizer Nano ZS90 (Malvern Instruments). Morphological topography and internal architecture were visualized using TEM and SEM. Elemental composition was examined by XPS and Energy Dispersive X‐ray Spectroscopy (EDS) mapping, confirming the homogeneous distribution of carbon (C), oxygen (O), nitrogen (N), phosphorus (P), and fluorine (F) within the nanostructure. Fluorine (F) was utilized as a specific compositional tracer to corroborate the co‐encapsulation of regorafenib.

To quantify the drug payload, the nanoparticles were demulsified using 1.0 mL of methanol containing 0.1% Triton X‐100 coupled with brief bath sonication. The extracted concentrations of regorafenib and erythrodiol were determined via a HPLC system (Agilent 1260 Infinity II) equipped with a reverse‐phase C18 analytical column (4.6 × 250 mm, 5 µm). For regorafenib, the isocratic mobile phase consisted of acetonitrile and 0.1% trifluoroacetic acid in water (60:40, v/v) at a constant flow rate of 1.0 mL/min, with UV detection set at 265 nm. Erythrodiol was independently quantified using a mobile phase of acetonitrile and water (85:15, v/v) at a flow rate of 1.0 mL/min with UV detection at 210 nm. The column compartment was maintained at 30 °C. The encapsulation efficiency (EE%) and loading capacity (LC%) were calculated using the following established equations:

$$\begin{array}{ccc} \mathrm{EE}\% & = & \text{Mass of encapsulated drug/Total mass of drug added}\\ & & \times \;100\% \end{array}$$

$$\begin{array}{ccc} \mathrm{LC}\% & = & \text{Mass of encapsulated drug/Total mass of nanoparticles}\\ & & \times \;100\% \end{array}$$

### 4.4 In Vitro pH‐Responsive Drug Release Kinetics

The temporal release profiles of regorafenib and erythrodiol from ApoBf‐R/E@NPs were quantitatively evaluated using the dynamic dialysis bag diffusion method under neutral and acidic conditions intended to represent extracellular and endolysosomal environments. Briefly, 1.0 mL of the purified nanoparticle suspension was tightly sealed within a regenerated cellulose dialysis membrane (molecular weight cut‐off: 3,500 Da) and completely immersed in 30.0 mL of the respective release media. To model neutral extracellular and acidic endolysosomal conditions, PBS (pH 7.4) and acetate buffer (pH 5.0), respectively, were used. Crucially, to strictly maintain sink conditions for the highly hydrophobic dual‐payloads and prevent premature compound precipitation, all release media were supplemented with 0.5% (v/v) Tween‐80. The dialysis assembly was maintained at 37 °C within a thermostatic shaking incubator at 100 rpm. At predefined time intervals (1, 2, 4, 8, 12, 24, and 48 h), a 1.0 mL aliquot of the external dialysate was withdrawn for HPLC quantification, and an equivalent volume of pre‐warmed, fresh medium was immediately replenished to maintain constant volume and sink dynamics. The cumulative release percentages were mathematically integrated over the 48 h evaluation window.

### 4.5 Cell Culture

Human GBM cell lines LN18 (Cat. No. CRL‐2610; RRID:CVCL_0392), U87MG (Cat. No. HTB‐14; RRID:CVCL_0022), T98G (Cat. No. CRL‐1690; RRID:CVCL_0556), and LN229 (Cat. No. CRL‐2611; RRID:CVCL_0393), together with the murine brain endothelial cell line bEnd.3 (Cat. No. CRL‐2299; RRID:CVCL_0170), and human umbilical vein endothelial cells (HUVECs; Cat. No. CRL‐1730; RRID:CVCL_2959), were obtained from ATCC. Murine GL261 cells (Cat. No. SNL‐174; RRID:CVCL_Y003) and the luciferase‐expressing derivative GL261‐Luc (Cat. No. SNL‐547) were obtained from Sunncell Biotechnology. U251MG cells (Cat. No. CL‐0237; RRID:CVCL_0021), normal human astrocytes (NHA; Cat. No. CP‐H122), and HMC3 human microglial cells (Cat. No. CL‐0620; RRID:CVCL_II76) were obtained from Procell Biotechnology. U87MG‐RFP cells (Cat. No. iCell‐0021a) were obtained from iCell Bioscience. Commercial human tumor cell lines were authenticated by the suppliers using short tandem repeat profiling where applicable. All cultures were routinely screened for mycoplasma contamination using a MycoBlue Mycoplasma Detector (Vazyme) and were negative before experimental use. Tumor cell lines were cultured in DMEM (Gibco, USA) supplemented with 10% FBS (Gibco) and 1% penicillin‐streptomycin. HUVEC, NHA, HMC3, and bEnd.3 cells were maintained under the vendors' recommended conditions. All cultures were incubated at 37°C in a humidified atmosphere containing 5% CO_2_ and passaged according to the corresponding culture conditions.

Patient‐derived GBM and GSC cultures. Fresh GBM specimens were obtained from patients undergoing surgical resection at the First Hospital of Jilin University after written informed consent. All procedures involving human specimens were approved by the Ethics Committee of the First Hospital of Jilin University (approval no. 19K127‐002), and the diagnosis of GBM was confirmed by neuropathological examination. Patient‐derived GBM cultures were anonymized and designated as GBM106, GBM132, GBM152, GBM114, and GBM178 to protect patient confidentiality. The corresponding clinical and neuropathological characteristics are summarized in Table S1. Participant demographic variables, including age and sex, were obtained from the institutional medical records at the time of surgical treatment and were recorded as documented in the clinical record; no demographic variables were inferred by the investigators. PBZ specimens were obtained from radiographically non‐tumorous brain regions outside the tumor core, jointly identified by the neuroradiologist and neurosurgeon according to preoperative imaging, anatomical location, and intraoperative neuronavigation. Fresh tumor tissues were mechanically minced, digested with 0.05% trypsin‐EDTA containing 0.2 mM EDTA at 37 °C for 30–45 min, and passed through a 70‐µm cell strainer to obtain single‐cell suspensions. Patient‐derived adherent GBM cells were maintained in DMEM/F12 supplemented with 10% FBS, 1 × GlutaMAX (Gibco, USA), 1 mM sodium pyruvate, and 1% penicillin–streptomycin and were used within five passages. For GSC establishment, freshly dissociated tumor cells were cultured as free‐floating neurospheres in low‐attachment vessels using serum‐free DMEM/F12 supplemented with 2% B27, recombinant human EGF (20 ng/mL, Gibco), bFGF (20 ng/mL, Gibco), 1× GlutaMAX, 1 mM sodium pyruvate, and 1% penicillin–streptomycin. Neurospheres were dissociated with Accutase for serial propagation and used within ten passages. GSC178 was established directly from freshly resected GBM178 tissue. The stem‐like phenotype of the GSC cultures was characterized by neurosphere growth and expression of CD133, NESTIN, SOX2, NANOG, and OLIG2. Patient‐derived cultures were maintained at 37 °C in a humidified atmosphere containing 5% CO_2_.

### 4.6 Cellular Uptake and Receptor‐Mediated Endocytosis

Internalization kinetics of ApoBf‐R/E@NPs were monitored across a 6‐h temporal window. For comparative uptake profiling, cells were exposed to PBS (control), E@NPs, R@NPs, R/E@NPs, or ApoBf‐R/E@NPs at an equivalent active‐agent concentration of 2 µM for 4 h. For the dual‐drug formulations, regorafenib and erythrodiol were maintained at a fixed 1:1 molar ratio. Intracellular fluorescence was visualized utilizing an Olympus IX73 inverted microscope and quantified via flow cytometry (BD FACS Celesta, USA). To assess the LDLR dependence of nanoparticle uptake, endogenous receptor expression was transiently silenced via specific siRNA targeting *LDLR* (in human lines) or *Ldlr* (in murine GL261 cells) using Lipofectamine 3000 (Invitrogen, USA) prior to nanoparticle exposure, followed by identical uptake quantification.

### 4.7 In Vitro Proliferation, Cytotoxicity, and Cell Cycle Profiling

Cellular metabolic viability was evaluated by CCK‐8 assay in established GBM cell lines, patient‐derived GBM106 cells, and GL261 cells. Briefly, cells (2 × 10^3^ cells/well) were exposed to the designated formulations across a concentration gradient (equivalent to 0.5–10.0 µM regorafenib) for 4 h. Following washout, cells were incubated in fresh complete medium for an additional 24 h before CCK‐8 measurement at 450 nm. Based on the derived dose‐response profiles, the respective half‐maximal inhibitory concentrations (IC50; approximately 0.8‐3 µM for human GBM lines and GBM106, and 4.0 µM for murine GL261 cells) were adopted as standardized working doses for all subsequent mechanistic evaluations. Unless otherwise specified, these cell‐specific working doses were used throughout the subsequent in vitro experiments, with all formulations matched by active‐agent equivalents and the R/E ratio maintained at 1:1 for dual‐drug formulations. For drug‐interaction analysis, free regorafenib and erythrodiol were combined at a fixed 1:1 molar ratio over the indicated concentration range in LN229 and patient‐derived GBM106 cells. Cells were subjected to the same 4‐h exposure and 24‐h recovery protocol described above, after which viability was determined by CCK‐8 assay. CI values were calculated using the Chou–Talalay method, with CI < 1, CI = 1, and CI > 1 indicating synergism, additivity, and antagonism, respectively.

For long‐term clonogenic survival, treated cells (1 × 10^3^ cells/well; exposed to their respective IC50 equivalent doses for 4 h followed by rigorous washout) were propagated in drug‐free basal medium for 7 days; resultant macroscopic colonies were fixed (4% paraformaldehyde), stained (0.1% crystal violet), and morphometrically quantified using ImageJ. Proliferative dynamics were traced using an EdU incorporation assay. Following a 4‐h treatment and a 24‐h post‐treatment recovery in fresh medium, cultures were pulsed with 50 µM EdU for 2 h, subjected to Apollo click‐chemistry labeling and Hoechst 33342 counterstaining, and analyzed via epifluorescence microscopy. Lytic membrane permeabilization was evaluated by quantifying extracellular LDH efflux using a colorimetric assay kit at the 24‐h mark. To analyze cell cycle progression, cells were treated at their respective IC50 equivalent doses for 4 h, washed, and allowed a 24‐h recovery to capture terminal cell cycle gridlock. Cells were subsequently harvested, fixed in ice‐cold 70% ethanol, and stained with a PI/RNase A solution. Cellular DNA content was acquired via flow cytometry, and phase distributions were modeled using FlowJo software.

### 4.8 Autophagic Flux and Intracellular Colocalization Assays

To systematically assess autophagosome‐lysosome fusion and proteolytic dynamics, multiparametric fluorescence imaging was conducted. LN229 and GL261 cells (2 × 10^5^ cells/well) established on glass coverslips (WHB BIO, China) were treated with the indicated formulations (at their respective IC50 equivalent doses) for 4 h. Following a 24‐h recovery in fresh medium, cells were fixed in 4% paraformaldehyde (30 min), permeabilized, and blocked simultaneously in 0.4% Triton X‐100 (Sigma–Aldrich) with 5% normal goat serum. Specimens were incubated overnight at 4°C with primary antibodies targeting the autophagosomal marker (LC3B; 1:200) and the lysosomal marker (LAMP1; 1:200). Following incubation with fluorophore‐conjugated secondary antibodies (Invitrogen; DyLight 488, #35552; DyLight 594, #35511; 1.5 h at 37°C), coverslips were mounted with DAPI. For dynamic flux monitoring, cells were transiently transfected with the mRFP‐GFP‐LC3 reporter plasmid via Lipofectamine 3000 for 24 h prior to treatment. Cells were exposed to formulations for 4 h, washed, and incubated in fresh medium for 24 h. Post‐treatment, cells were fixed, and autophagosomes (yellow puncta) versus autolysosomes (red puncta) were quantified across ≥ 3 random fields using ImageJ. Concurrently, lysosomal proteolytic activity was quantified by pulse‐labeling cells with 10 µg/mL DQ‐BSA Red for 1 h at 37 °C. After removing the labeling medium, cells were treated for 4 h, allowed a 24‐h recovery, fixed, and de‐quenched fluorescence was captured using confocal (Olympus SpinSR10) or inverted (Olympus IX73) microscopes.

### 4.9 Cell Transfection

Transient transfection was performed using Lipofectamine 3000 according to the manufacturer's instructions. Cells were seeded one day before transfection and grown to 60%–80% confluence. For mRFP‐GFP‐LC3 transfection, plasmid DNA was diluted in Opti‐MEM and mixed with P3000 reagent, followed by incubation with diluted Lipofectamine 3000 to form DNA‐lipid complexes. For gene silencing experiments, species‐matched siRNA duplexes targeting LDLR/Ldlr or ABCA1/Abca1 were diluted in Opti‐MEM and mixed with Lipofectamine 3000 to generate siRNA‐lipid complexes. The complexes were added to cells and incubated for 4–6 h, after which the medium was replaced with fresh complete medium. Cells were harvested or imaged 24–48 h after transfection for subsequent analyses. The siRNA sequences for human LDLR and ABCA1, and mouse Ldlr and Abca1, are listed in Table S2.

For autophagic flux analysis, cells transfected with mRFP‐GFP‐LC3 were observed under a confocal microscope to detect autophagosomes and autolysosomes. For gene silencing experiments, cells transfected with LDLR/Ldlr or ABCA1/Abca1 siRNAs were collected at the indicated time points, and knockdown efficiency was confirmed by Western blot analysis.

### 4.10 In Vitro Endothelial Tube Formation Assay

The antiangiogenic properties of the nanoplatform were evaluated in vitro by assessing the morphological differentiation of HUVECs into capillary‐like networks. Briefly, pre‐chilled 96‐well plates were coated with 50 µL of Matrigel (Corning, #354262) per well and incubated at 37 °C for 30 min to allow matrix polymerization. HUVECs (2 × 10^4^ cells/well) treated with the indicated formulations at an equivalent active‐agent concentration of 2 µM were then seeded onto the solidified Matrigel. Following a 6‐h incubation at 37°C, the three‐dimensional tubular networks were photographed using an inverted bright‐field microscope (Olympus IX73). Quantitative architectural indices, specifically total tube length and the number of branching points, were analyzed utilizing the Angiogenesis Analyzer plugin within ImageJ software.

### 4.11 Immunoblotting

For baseline expression profiling, untreated established GBM cell lines, patient‐derived GBM cells, GSC cultures, and freshly collected human specimens were lysed directly in RIPA buffer supplemented with protease and phosphatase inhibitors. For treatment‐response analyses, established GBM cells, patient‐derived GBM132 cells, and GSC178 cultures were exposed to the indicated formulations for 4 h, washed, and maintained in fresh culture medium for the assay‐specific recovery period (generally 12–24 h). Whole‐cell lysates were then collected in RIPA buffer supplemented with protease and phosphatase inhibitors. For in vivo molecular profiling, excised tumor tissues were immediately snap‐frozen, mechanically homogenized, and lysed in ice‐cold RIPA buffer. Homogenates were centrifuged (12 000 × g, 30 min, 4°C), and equivalent protein quantities were resolved via SDS‐PAGE and transferred to PVDF membranes (Millipore, #IPVH00010). Membranes were blocked with 5% non‐fat milk in Tris‐buffered saline with 0.1% Tween‐20 (TBST) for 2 h, followed by overnight incubation at 4°C with designated primary antibodies. After standard TBST washes, membranes were incubated with HRP‐conjugated secondary antibodies (1:5,000) for 1.5 h. Chemiluminescent signals were detected using an enhanced chemiluminescence (ECL) kit (Biosharp, #BL5965B), captured via a contact‐area chemiluminescence imager (e‐Blot, China), and quantified using ImageJ. Target protein expression was normalized to the respective loading controls.

### 4.12 Apoptosis, Oxidative Stress, and Bioenergetic Profiling

To evaluate treatment‐induced cellular stress and death pathways, multiparametric flow‐cytometric and fluorometric analyses were performed in established GBM cells and, where indicated, patient‐derived GBM132 cells. Cells (2 × 10^5^ cells/well) were treated with the designated formulations (at respective IC50 equivalents) for 4 h, thoroughly washed to remove extracellular nanoparticles, and incubated in fresh complete medium for an additional 12–24 h depending on the specific endpoint (12 h for rapid ATP/ROS metabolic shifts, and 24 h for mitochondrial membrane potential and apoptosis). For apoptosis quantification, both adherent and floating fractions were harvested, washed with ice‐cold PBS, and double‐stained with Annexin V‐FITC and PI for 15 min in the dark. The same workflow was used for patient‐derived GBM132 cells. Apoptotic populations were acquired via flow cytometry (BD FACS Celesta) and partitioned via quadrant gating in FlowJo. For the BBB‐associated tumor‐normal co‐culture experiments, apoptosis was assessed using Annexin V/DAPI staining. U87MG‐RFP cells were distinguished from NHA or HMC3 cells according to intrinsic RFP fluorescence, and apoptotic fractions were quantified independently within the RFP‐positive tumor‐cell and RFP‐negative non‐tumor‐cell gates. Concurrently, intracellular reactive oxygen species (ROS) and ΔΨm were assessed using DCFH‐DA (10 µM) and JC‐1 ratiometric probes, respectively. Post‐treatment, cells were incubated with the probes for 30 min at 37°C, washed to remove extracellular fluorophores, and analyzed via flow cytometry and fluorescence microscopy. To determine energetic deficit, intracellular ATP levels were quantified in LN229, GL261, and patient‐derived GBM132 cells, as indicated in the corresponding experiments. Clarified cell lysates were reacted with the ATP luminescence reagent, and ATP content was normalized to total protein concentration. For metabolic complementation experiments, GBM132 cells and the indicated established GBM models were additionally subjected to cholesterol supplementation, 3‐MA treatment, or their combination before ATP measurement.

### 4.13 Lipid Homeostasis and Transporter Degradation Kinetics

Intracellular lipid homeostasis was assessed in patient‐derived GBM132 cells, with parallel analyses performed in LN229 and GL261 cells where indicated. Total cholesterol, free cholesterol, and triglycerides were quantified 24 h after the 4‐h transient treatment using the corresponding enzymatic assay kits, with signals normalized to total protein content. Lipid‐droplet imaging was performed in LN229 and GL261 cells using BODIPY 493/503 staining followed by fluorescence microscopy and image‐based quantification. To assess ABCA1 stability, LN229 and GL261 cells were subjected to cycloheximide chase analysis. Following the indicated treatment, cells were exposed to 20 µM cycloheximide for 0, 6, or 12 h, and residual ABCA1 abundance was quantified by immunoblotting relative to the corresponding t = 0 value.

### 4.14 Public‐Dataset Bioinformatics Analysis and Transcriptomic Sequencing

Publicly available transcriptomic datasets were additionally interrogated to characterize LDLR expression across bulk GBM tissues, glioma stem‐like cells, anatomical tumor regions, molecular subtypes, malignant cell states, and major cellular compartments. For the bulk tumor–normal comparison, normalized LDLR expression values from GBM and Genotype‐Tissue Expression (GTEx) normal‐brain cohorts were retrieved from UCSC Xena Toil hub. TCGA and GTEx were jointly processed via the Toil pipeline, RSEM gene‐level transcripts per million (TPM) values, stored as log2(TPM+0.001), were used, and the groups were compared using a two‐sided Mann‐Whitney U test. LDLR expression in GSCs and normal brain was analyzed using raw CEL files from GSE20736 and GSE30563, which were jointly normalized with the RMA algorithm (R package affy 1.88.0 with hgu133plus2cdf annotation; R 4.5.0). Groups were compared with the two‐sided Wilcoxon rank‐sum test. Spatially resolved expression data were obtained from the Ivy Glioblastoma Atlas Project and stratified into leading edge (LE), infiltrating tumor (IT), cellular tumor (CT), pseudopalisading cells around necrosis (PAN), perinecrotic zone (PNZ), microvascular proliferation (MVP), and hyperplastic blood vessel (HBV) compartments. Molecular subtype analyses were performed on CGGA325 RNA‐seq cohorts restricted to WHO grade IV tumors (n = 139). Samples were classified into Verhaak subtypes (Classical, Mesenchymal, Proneural, Neural) and Wang subtype (Classical/Mesenchymal/Proneural) by single‐sample gene‐set enrichment (ssGSEA). LDLR was compared across subtypes with the Kruskal‐Wallis and pairwise Mann‐Whitney tests with FDR correction.

For the single‐cell analysis, we interrogated the IDH‐mutant astrocytoma single‐cell RNA‐sequencing dataset reported by Tang et al. (Cancer Cell, 2025). As described in the original study, we built on previously defined transcriptional states in IDH‐mutant astrocytoma and re‐analyzed malignant cells by unsupervised clustering. Six tumor cell states were identified and annotated by canonical markers: neural‐progenitor‐like (NPC‐like), MES‐like, AC‐like, OC‐like, G1/S, and G2/M. LDLR expression was compared across the four non‐cycling tumor cell states (NPC‐like, MES‐like, AC‐like, and OC‐like). For each state, mean normalized LDLR expression and the percentage of LDLR‐positive cells were calculated, using the processed (normalized) expression values provided by the original study and analyzed with Seurat (version 5.0). Data processing and visualization were performed in R 4.5.0 (packages: affy 1.88.0, hgu133plus2cdf, ggplot2, dplyr) and Python 3.13 (pandas, numpy, scipy, statsmodels). Statistical comparisons were conducted using two‐sided Mann‐Whitney U / Wilcoxon rank‐sum tests and Kruskal‐Wallis tests, with Benjamini‐Hochberg FDR correction for multiple comparisons where applicable.

Total RNA was isolated from cells subjected to the indicated treatments using TRIzol reagent (Thermo Fisher Scientific, #15596018CN). Library preparation and high‐throughput transcriptomic sequencing (Illumina platform) were subsequently performed by OBiO Technologies (Shanghai, China) utilizing three independent biological replicates (n = 3) per cohort. Raw and processed transcriptomic data have been deposited in the NCBI Gene Expression Omnibus (GEO) database. Differential gene expression analysis was performed using the DESeq2 R package, with statistical significance defined by an adjusted *p*‐value (padj) < 0.05 and an absolute log2 fold change > 1.0. Pathway enrichment analyses, including KEGG and GO, were executed utilizing the clusterProfiler package. GSEA was applied to systematically evaluate biological signatures.

### 4.15 In Vitro BBB Transendothelial Transport and Tumor‐Normal Co‐Culture Assays

To evaluate transendothelial delivery, an in vitro BBB model was established using Transwell inserts (0.4 µm pore size; Corning). bEnd.3 endothelial cells (1 × 10^5^ cells/insert) were seeded in the apical chamber and cultured to confluence, while LN229 or GL261 cells (5 × 10^4^ cells/well) were seeded in the basolateral chamber. After equilibration, the apical compartment was exposed to the indicated formulations for 4 h. Formulation‐associated fluorescence in the recipient GBM cells was assessed by fluorescence microscopy and flow cytometry, and the biological consequence of transendothelial delivery was evaluated by clonogenic survival.

For BBB‐associated tumor–normal co‐culture experiments, U87MG cells stably expressing RFP were co‐cultured with NHA or HMC3 cells at a 1:1 ratio in the basolateral compartment beneath a confluent bEnd.3 monolayer. Following apical exposure to the indicated formulations, the basolateral cells were collected and analyzed by flow cytometry. U87MG‐RFP and non‐tumor cells were resolved on the basis of RFP fluorescence, allowing apoptosis to be quantified separately in the two cellular populations as described above.

### 4.16 Tumorsphere Formation and Patient‐Derived GSC Viability Assays

To assess the capacity for anchorage‐independent growth and the maintenance of stem‐like properties, a three‐dimensional tumorsphere assay was performed. LN229 and GL261 cells were pretreated with the designated formulations for 4 h. Following an overnight recovery, the cellular cohorts were suspended in complete DMEM containing 0.5% agarose and overlaid onto a solidified 1% agarose base layer in 6‐well plates. The matrices were hydrated with complete medium and incubated for 7 days. Tumorsphere formation was subsequently visualized and morphometrically quantified using bright‐field microscopy.

For patient‐derived GSC assays, GSC178 neurospheres were exposed to the indicated formulations at an equivalent active‐agent concentration of 2 µM for 4 h, washed to remove extracellular formulations, and subsequently maintained in fresh serum‐free GSC medium. At 24 h after washout, neurosphere viability was assessed using the Beyo3D Calcein/PI Cell Viability Assay Kit (Beyotime, China) according to the manufacturer's instructions. Calcein and PI fluorescence were used to visualize viable and membrane‐compromised cells, respectively. In parallel, GSC178 neurospheres were monitored by bright‐field imaging using an Incucyte SX5 live‐cell analysis system (Sartorius), and surviving tumorspheres were quantified relative to the corresponding control.

### 4.17 Tumor‐Immune In Vitro Co‐Culture System

To delineate the immunomodulatory crosstalk between treated glioma cells and the immune compartment, a primary co‐culture system was established. Primary splenocytes and bone marrow‐derived cells were isolated from healthy syngeneic mice under aseptic conditions, filtered through a 70 µm mesh, and erythrocyte‐depleted. GL261 cells were pretreated with the indicated formulations, including carrier‐matched unloaded ApoBf@NPs where specified, for 4 h (approximately 4.0 µM IC50 equivalent dose) and thoroughly washed to remove uninternalized nanoparticles, and were co‐cultured with these primary immune fractions at a predetermined effector‐to‐target (E:T) cell ratio of 10:1 for 48 h. Bone marrow‐derived fractions were used for DC and macrophage profiling, whereas splenocytes were used for T‐cell and Treg analyses. The immune compartments were subsequently harvested, labeled with specific fluorophore‐conjugated antibodies, and phenotypically profiled via a BD FACS Celesta flow cytometer with downstream analysis in FlowJo.

### 4.18 Animal Subjects and In Vivo Syngeneic Tumor Models

Female C57BL/6 mice (5 weeks old; Vital River Laboratory Animal Technology, China) were housed under specific pathogen‐free (SPF) conditions. Female cohorts were exclusively utilized to preclude aggression‐induced injuries and ensure behavioral compatibility during the long‐term group housing required for robust survival and abscopal monitoring. All animal protocols were conducted in strict compliance with institutional guidelines and explicitly approved by the Animal Ethics and Use Committee of the First Hospital of Jilin University (approval no. 20240222‐08). An in vivo Matrigel plug assay and three syngeneic GL261 tumor models were used to evaluate angiogenesis, therapeutic efficacy, and immune responses. Within each in vivo model, all drug‐loaded formulations were administered at matched active‐agent equivalents, with regorafenib and erythrodiol maintained at a 1:1 molar ratio in the dual‐drug formulations.

Matrigel plug angiogenesis model: To quantify the antiangiogenic efficacy in vivo, a Matrigel plug assay was conducted. Unpolymerized Matrigel (500 µL) containing the respective nanoparticle formulations (formulated to deliver an absolute localized dose equivalent to 100 µg regorafenib per plug) was injected subcutaneously into the abdominal midline of C57BL/6 mice. The injected matrix rapidly polymerized at body temperature to form a solid bio‐scaffold. Following 14 days of continuous host vascular infiltration, the mice were euthanized, and the Matrigel plugs were carefully excised. The recovered implants were macroscopically photographed to document gross vascularization and erythema. Subsequently, to provide a rigorous quantitative assessment of neovascularization, the hemoglobin content within the implants was evaluated. The excised plugs were homogenized in deionized water, and the hemoglobin concentration was determined colorimetrically utilizing Drabkin's reagent (Merck, #D5941) according to the manufacturer's protocol. The absorbance of cyanmethemoglobin was recorded at 540 nm using a microplate reader, and the final hemoglobin levels were normalized to the total mass of the respective Matrigel plugs.

Orthotopic GBM model: Six‐week‐old mice were anesthetized and stereotactically inoculated with luciferase‐expressing GL261 (GL261‐Luc) cells (1 × 10^5^ cells suspended in 2 µL of sterile PBS) into the right striatum (coordinates from bregma: 0.5 mm anterior, 2.5 mm lateral, 3.0 mm depth). Following a 7‐day engraftment, cohorts were randomized and administered the designated formulations intravenously (i.v.) via the tail vein at a standardized equivalent dose of 5.0 mg/kg for regorafenib and 4.6 mg/kg for erythrodiol (strictly maintaining the active equimolar stoichiometry; injection volume: 100 µL) every other day for five cycles. In vivo biodistribution and cranial formulation‐associated fluorescence were monitored longitudinally using an AniView Kirin imaging system (BLT, China).

Bilateral abscopal model: To evaluate systemic immune activation, bilateral syngeneic GL261 tumors were established by subcutaneously inoculating GL261 cells into both murine flanks. Primary right‐flank lesions received intratumoral (i.t.) interventions (dose equivalent to 2.5 mg/kg regorafenib and 2.3 mg/kg erythrodiol in a concentrated 20 µL injection volume; administered every other day for five cycles), while contralateral left‐flank tumors were left untreated to serve as systemic surrogates. Tumor volumetrics across both sites were monitored concurrently to delineate localized versus distal immune‐mediated inhibition.

Postsurgical recurrence model: Mice bearing unilateral subcutaneous GL261‐Luc syngeneic tumors (500‐600 mm^3^) underwent subtotal resection with macroscopic residual disease intentionally retained. Residual GL261‐Luc burden was confirmed by BLI before initiation of postoperative treatment. Following a 7‐day postoperative recovery, cohorts were subjected to the specified systemic regimens via intravenous (i.v.) tail vein injection (equivalent to 5.0 mg/kg regorafenib and 4.6 mg/kg erythrodiol in 100 µL; administered every other day for five cycles). Tumor recurrence and overall survival were subsequently monitored. Across all regimens, systemic toxicity was longitudinally tracked via body weight profiling every 48 h.

### 4.19 Histopathological and Intratumoral Protein Profiling

To evaluate treatment‐induced intratumoral alterations and spatial protein expression, histological analyses of the tumor microenvironment were performed. Upon completion of the in vivo regimens, tumor‐bearing brains and subcutaneous lesions were excised, immersion‐fixed in 4% paraformaldehyde (24 h), and paraffin‐embedded (5 µm sections). H&E staining was used to assess tumor histoarchitecture and treatment‐associated morphological changes. Apoptotic DNA fragmentation was assessed using a TUNEL assay kit (Beyotime, China) according to the manufacturer's protocol. For spatial mechanistic profiling, IHC and immunofluorescence staining were conducted. Sections were deparaffinized, rehydrated, and subjected to heat‐induced epitope retrieval in citrate buffer (pH 6.0). Following a 30‐min blockade (3% BSA), sections were incubated overnight at 4 °C with specific primary antibodies. For IHC, HRP‐conjugated secondary antibodies were applied, followed by 3,3′‐diaminobenzidine (DAB) chromogenic development. For IF, sections were incubated with fluorophore‐conjugated secondary antibodies, counterstained with DAPI, and imaged via confocal laser‐scanning microscopy (Olympus SpinSR10). Quantitative analysis of positive cellular populations was executed using ImageJ.

### 4.20 Systemic Biosafety and Toxicity Evaluation

To comprehensively monitor the systemic safety profile of the therapeutic regimens, both histological and serological evaluations were conducted. Off‐target major organs (brain, heart, liver, spleen, lung, and kidney) were harvested, processed into paraffin sections, and stained with H&E to screen for architectural abnormalities, inflammatory infiltrates, or tissue damage. Concurrently, whole blood was harvested via the retro‐orbital plexus from anesthetized mice. Serum biochemical analysis included hepatic parameters (ALB, ALP, ALT, AST, DBil, TBil, and TP), renal parameters (Crea, UA, and UREA), myocardial and tissue‐injury markers (CKMB and LDH), pancreatic enzymes (AMY and LIP), glucose and lipid metabolic indices (GLU, CHOL, HDL, LDL, and TG), and the inflammatory marker CRP.

### 4.21 In Vivo Immune‐Cell and Cytokine Profiling

Fresh tumor tissues, spleens, and bone marrow were collected at the designated experimental endpoints and processed immediately for flow‐cytometric analysis. Tumor tissues were mechanically minced followed by filtration through a 70‐µm cell strainer to obtain single‐cell suspensions. Splenocytes were prepared by mechanical dissociation through a 70‐µm strainer, whereas bone marrow cells were flushed from the femurs and tibias with RPMI‐1640 medium. Erythrocytes were removed using red blood cell lysis buffer where required.

Following washing, cells were stained with the indicated fluorophore‐conjugated antibodies and analyzed using a BD FACS Celesta flow cytometer, with downstream analysis performed in FlowJo. For tumor‐derived immune‐cell profiling, singlet viable cells were first gated on CD45^+^ leukocytes. Mature dendritic cells were identified as CD11c^+^CD80^+^CD86^+^ cells. Macrophages were gated as CD11b^+^F4/80^+^ cells, followed by identification of iNOS^+^ M1‐like and CD206^+^ M2‐like populations. T lymphocytes were gated as CD3^+^ cells and subsequently resolved into CD8a^+^ and CD4^+^ subsets; regulatory T cells were identified within the CD3^+^CD4^+^ compartment as CD25^+^Foxp3^+^ cells. Bone marrow‐derived cells were used for DC and macrophage profiling, whereas splenocytes were used for CD8^+^, CD4^+^, and regulatory T‐cell analyses. For analysis of memory‐associated T‐cell populations in recurrent tumors, CD8^+^ and CD4^+^ T‐cell compartments were first identified within the CD45^+^CD3^+^ population, after which T_CM_ were defined as CD44^+^CD62L^+^ and T_EM_ as CD44^+^CD62L^−^.

For systemic cytokine analysis, serum was isolated from retro‐orbital blood collected at the indicated endpoint. Concentrations of IL‐2, IL‐6, IL‐10, TGF‐β, TNF‐α, IFN‐γ, granzyme B, and perforin were determined using commercial ELISA kits according to the manufacturers’ protocols. Absorbance was measured using a Tecan Spark multimode microplate reader, and cytokine concentrations were calculated from analyte‐specific standard curves.

### 4.22 Statistical Analysis

Statistical analyses and data visualization were performed using GraphPad Prism 10.0. Unless otherwise indicated, quantitative data are presented as mean ± standard deviation (SD), with n representing independent biological replicates for in vitro experiments or individual animals for in vivo experiments. Technical replicates, where performed, were averaged within each biological replicate and were not treated as independent observations. No statistical method was used to predetermine sample size; sample sizes were chosen based on prior experience with the corresponding experimental models and established experimental practice. Animals were randomly allocated to treatment groups, and endpoint assessments, including histological quantification, were performed blinded to group allocation where applicable.

Comparisons between two independent groups were performed using an unpaired two‐tailed Student's t‐test. Single‐endpoint comparisons among three or more independent groups were analyzed by one‐way analysis of variance (ANOVA) followed by Tukey's multiple‐comparisons test. Longitudinal measurements repeatedly acquired from the same animals, including cranial fluorescence, tumor bioluminescence, tumor volume, and body weight, were analyzed by two‐way repeated‐measures ANOVA with treatment and time as factors, followed by Šídák's multiple‐comparisons test. For the paired comparison of post‐resection and recurrent tumor bioluminescence, treatment group and disease stage were analyzed as the two factors in a two‐way repeated‐measures ANOVA, followed by Šídák's multiple‐comparisons test. Survival distributions were estimated using the Kaplan–Meier method and compared using the log‐rank (Mantel‐Cox) test.

Densitometric values displayed beneath representative immunoblot bands were normalized to β‐actin and expressed relative to the indicated reference sample, which was set to 1.00. Where statistical analysis of immunoblot data was performed, independent biological replicates rather than individual band measurements were used as the unit of analysis. Statistical procedures applied to public transcriptomic datasets are described separately in the corresponding bioinformatics section. All statistical tests were two‐sided, and p < 0.05 was considered statistically significant. Significance levels are denoted as ^*^ *p* < 0.05, ^**^ *p* < 0.01, ^***^ *p* < 0.001, ^****^ *p* < 0.0001; ns, not significant.

## Author Contributions

**Yining Jiang**: Investigation, data curation, formal analysis, methodology, validation, visualization, funding acquisition, writing – original draft. **Wenzhuo Yang**: Data curation, formal analysis, software, visualization. **Wenting Chen**: Investigation, methodology, validation. **Jing Zuo**: Investigation, validation. **Lin Pan**: Resources, investigation. **Sheng Zhong**: Formal analysis, software. **Ren Geng**: Investigation, data curation. **Yao Zhao**: Investigation, validation. **Zijian Chen**: Investigation, validation. **Canhua Huang**: Conceptualization, project administration, supervision, writing – review and editing. **Yang Bai**: Resources, supervision, writing – review and editing. **Liyan Zhao**: Resources, supervision, writing – review and editing. **Yunqian Li**: Conceptualization, funding acquisition, project administration, supervision, writing – review and editing.

## Conflicts of Interest

The authors declare no conflicts of interest.

## Supporting information




**Supporting File**: advs78189‐sup‐0001‐SuppMat.docx.

## Data Availability

The data that supports the findings of this study are available in the supplementary material of this article.
