# Supplementary material for: Low‐Density Lipoprotein Receptor‐Targeted Biomimetic Nanoplatform Suppresses Glioblastoma Stemness and Remodels Antitumor Immunity via Dual Metabolic Disruption
Source: Adv Sci (Weinh). 2026 Oct 11:e78189. Online ahead of print. doi: 10.1002/advs.78189 (PMC13654861; doi:10.1002/advs.78189)
Supplement: Supplementary file 1 [file ADVS-9999-e78189-s001.docx]

**Supporting Information**

**Low-Density Lipoprotein Receptor-Targeted Biomimetic Nanoplatform Suppresses Glioblastoma Stemness and Remodels Antitumor Immunity via Dual Metabolic Disruption**

Yining Jiang^a^, Wenzhuo Yang^c^, Wenting Chen^b^, Jing Zuo^b^, Lin Pan^e^, Sheng Zhong^c^, Ren Geng^a^, Yao Zhao^a^, Zijian Chen^c^, Canhua Huang^b,f,g^, Yang Bai^a^*^*^*, Liyan Zhao^d^*^*^*, Yunqian Li^a^*^*^*

*^a^ Department of Neurosurgery, Translational Medicine Research Institute, First Hospital of Jilin University, Changchun 130021, P.R. China.*

*^b^ Department of Biotherapy, Institute of Oxidative Stress Medicine, Cancer Center and State Key Laboratory of Biotherapy, West China Hospital and West China School of Basic Medical Sciences and Forensic Medicine, Sichuan University, Chengdu, 610041, P.R. China.*

*^c^ Department of Neurosurgery, State Key Laboratory of Oncology in South China, Collaborative Innovation Center for Cancer Medicine, Sun Yat-sen University Cancer Center, Guangzhou, 510060, P.R. China.*

*^d^ Department of Blood Transfusion, Second Hospital of Jilin University, Changchun, Jilin 130041, P.R. China.*

*^e^ Department of Neurology, Neuroscience Research Center, The First Hospital of Jilin University, Changchun 130021, P.R. China.*

*^f^ Frontiers Medical Center, Tianfu Jincheng Laboratory, Chengdu, 610041, P.R. China.*

*^g^ Department of Surgical Oncology, The First Affiliated Hospital of Kunming Medical University, Kunming, 650032, P.R. China.*

*^*^Corresponding authors. E-mail: Yunqian Li: yunqian@jlu.edu.cn. Liyan Zhao:* *zhaoliy@jlu.edu.cn. Yang Bai: baiyang0221@jlu.edu.cn.*

**Fig. S1.** Optimization of the protein corona and quantitative chromatographic methodology for ApoBf-R/E@NPs. (A,B) Titration of the ApoB-fragment-to-R/E@NPs mass ratio, monitored by variations in hydrodynamic diameter (A) and zeta potential (B), establishing 2.0 μg/mg as the optimal formulation parameter. (C) Hydrodynamic size distribution of the undecorated, bare R/E@NPs. Inset: macroscopic visualization of the Tyndall effect. (D-G) Size-exclusion chromatography (SEC) elution profiles. The synchronous co-elution of the fluorescent lipid core (FITC) and the protein corona (A280) within the void volume confirms the stable anchorage of ApoB-fragments, distinguishing intact nano-assemblies from the delayed elution of free peptides. (H-K) Matrix-matched high-performance liquid chromatography (HPLC) calibration curves constructed for the quantification of erythrodiol (H,I) and regorafenib (J,K). To minimize matrix effects, independent equations were established under acidic/endolysosomal-mimicking (pH 5.0) and physiological (pH 7.4) conditions. *A* represents the chromatographic peak area (mAU·s), and *C* denotes the analyte concentration (μg/mL).

**Fig. S2.** Supplementary analysis of LDLR expression heterogeneity and ApoBf-mediated uptake. (A) LDLR expression across Wang-defined classical, mesenchymal, and proneural GBM transcriptional subtypes. (B,C) Uptake of R/E@NPs and ApoBf-R/E@NPs in GL261 cells assessed by fluorescence imaging (B) and quantification of formulation-associated FITC-positive cells normalized to total cell number (C). Scale bar, 50 μm. (D,E) Time-dependent uptake of ApoBf-R/E@NPs in GL261 cells over 0-6 h assessed by fluorescence imaging (D) and flow cytometry with corresponding quantitative analysis (E). Scale bar, 50 μm. (F) Flow-cytometric analysis and corresponding quantification of ApoBf-R/E@NP-associated fluorescence in control and Ldlr-silenced GL261 cells.

**Fig. S3.** Additional evaluation of ApoBf-R/E@NP-mediated antitumor activity and tumor selectivity. (A-D) Cell viability assessed by CCK-8 assay in LN18 (A), T98G (B), normal human astrocytes (NHA; C), and HMC3 microglial cells (D) following treatment with the indicated formulations. (E) Fixed-ratio combination-index (CI) analysis of regorafenib and erythrodiol in LN229 cells at a 1:1 molar ratio over the indicated concentration range. The dashed line denotes CI = 1; CI < 1 indicates synergism. (F) Schematic of the in vitro BBB transwell model with bEnd.3 endothelial cells in the upper chamber and GL261 cells in the lower chamber. (G,H) Transendothelial formulation delivery assessed by fluorescence imaging (G) and flow cytometry (H) in recipient GL261 cells. Scale bar, 50 μm. (I,J) Clonogenic survival of GL261 cells following transendothelial delivery (I) and corresponding quantification (J). (K) Schematic of the BBB-associated tumor–normal cell co-culture model comprising bEnd.3 endothelial cells, U87MG-RFP cells, and HMC3 microglial cells. (L) Representative Annexin V/DAPI flow-cytometric profiles of separately gated U87MG-RFP and HMC3 populations following treatment with the indicated formulations. (M,N) Quantification of apoptotic U87MG-RFP cells (M) and HMC3 cells (N). (O,P) Representative EdU incorporation images in GL261 cells (O) and quantification of EdU-positive cells (P). Scale bar, 50 μm. (Q,R) Clonogenic survival assay in GL261 cells (Q) and corresponding colony quantification (R). (S) LDH release from GL261 cells following treatment with the indicated formulations. (T) Immunoblot analysis of CDK2, Cyclin E2, p-cdc2 (Thr161), Cyclin B1, and Cyclin A2 in GL261 cells.

**Fig. S4.** Supplementary evaluation of autophagic blockade and lysosomal dysfunction. (A, B) Confocal imaging (A) and colocalization analysis (B) of LC3B and LAMP1 in GL261 cells. Scale bar, 10 μm. (C, D) Tandem reporter mRFP-GFP-LC3 assays for autophagic flux in GL261 cells. Scale bar, 10 μm. (E, F) DQ-BSA fluorescence assessment of lysosomal proteolytic activity in GL261 cells. Scale bar, 50 μm. (G) Immunoblotting of LC3B-II, SQSTM1/p62, and PSAT1 accumulation in GL261 cells. (H, I) Clonogenic survival and cell viability assays with 3-MA treatment in GL261 cells. (J, K) Cell viability and survival after CQ-mediated late-stage autophagy inhibition in GL261 cells. (L) Immunoblotting of autophagy-related proteins after 3-MA or CQ treatment in GL261 cells.

**Fig. S5.** Mitochondrial stress and apoptotic responses to ApoBf-R/E@NPs in GL261 and patient-derived GBM132 cells. (A,B) Intracellular ROS accumulation in GL261 cells assessed by DCFH-DA fluorescence imaging (A) and flow cytometry (B). Scale bar, 50 μm. (C,D) Mitochondrial membrane potential (ΔΨm) in GL261 cells assessed by JC-1 fluorescence imaging (C) and flow cytometry (D). Scale bar, 50 μm. (E,F) Annexin V/PI flow-cytometric analysis of apoptosis in GL261 cells (E) and corresponding quantification (F). (G,H) Annexin V/PI flow-cytometric analysis of apoptosis in patient-derived GBM132 cells (G) and corresponding quantification (H). (I,J) Immunoblot analysis of apoptosis-associated proteins in GL261 cells (I) and patient-derived GBM132 cells (J), including Bax, Bcl-2, Caspase-3, cleaved Caspase-3, PARP-1, and cleaved PARP-1.

**Fig. S6.** ABCA1 stabilization, lipid depletion, and ATP loss in LN229 and GL261 glioma cells. (A) Immunoblot analysis of ABCA1 abundance in GL261 cells following treatment with the indicated formulations. (B,C) Cycloheximide (CHX) chase analysis of ABCA1 stability in GL261 cells, showing representative immunoblots at 0, 6, and 12 h (B) and quantification of the remaining ABCA1 protein (C). (D,E) Biochemical quantification of total cholesterol in LN229 (D) and GL261 (E) cells. (F,G) Biochemical quantification of free cholesterol in LN229 (F) and GL261 (G) cells. (H,I) Biochemical quantification of triglycerides in LN229 (H) and GL261 (I) cells. (J,K) Fluorescence imaging of intracellular lipid droplets in GL261 cells (J) and quantitative analysis of fluorescence intensity (K). Scale bar, 50 μm. (L,M) Intracellular ATP levels in LN229 (L) and GL261 (M) cells following treatment with the indicated formulations. (N,O) Functional complementation of ATP levels in LN229 (N) and GL261 (O) cells following cholesterol supplementation (+CHO), inhibition of autophagy initiation with 3-MA (+3-MA), or their combination (+CHO+3-MA).

**Fig. S7.** Additional transcriptomic and biochemical analyses of immune-associated programs and survival signaling. (A,B) GSEA of natural killer cell-mediated cytotoxicity (A) and apoptotic DNA fragmentation (B) in ApoBf-R/E@NP-treated versus control LN229 cells. Each GSEA panel includes an inset heatmap of representative genes contributing to the corresponding enriched signature. (C,D) Immunoblot analysis of the PI3K/AKT/mTOR (C) and RAF1/MEK/ERK/JNK (D) signaling cascades in GL261 cells following treatment with the indicated formulations. (E) Immunoblot verification of siRNA-mediated Abca1 knockdown in GL261 cells. (F,G) Immunoblot analysis of PI3K/AKT/mTOR (F) and RAF1/MEK/ERK/JNK (G) signaling in GL261 cells treated with the indicated formulations in the presence or absence of Abca1 silencing.

**Fig. S8.** Additional analyses of glioma stemness and immune-cell remodeling. (A) Representative bright-field images of GL261 tumorspheres following treatment with the indicated formulations. Scale bar, 100 μm. (B) Representative flow-cytometric profiles of the CD133^+^ fraction in LN229 cells. (C,D) Representative flow-cytometric profiles of CD133^+^ GL261 cells (C) and corresponding quantification (D). (E) Immunoblot analysis of NOTCH1, OCT4, KLF4, NANOG, and SOX2 in GL261 cells. (F,G) Immunoblot analysis of cellular HMGB1 and CRT abundance in whole-cell lysates from LN229 (F) and GL261 (G) cells. (H,I) Representative flow-cytometric analysis of CD3^+^CD4^+^ T cells following co-culture with treated GL261 cells (H) and corresponding quantification (I). Densitometric values shown below immunoblot bands were normalized to β-actin and expressed relative to the corresponding control.

**Fig. S9.** Physicochemical and biological evaluation of unloaded ApoBf@NPs. (A) Representative TEM image of unloaded ApoBf@NPs. Scale bar, 30 nm. (B) Hydrodynamic size distribution of unloaded ApoBf@NPs measured by DLS, showing a Z-average diameter of 157.3 nm. (C) CCK-8 analysis of LN229 cell viability following treatment with unloaded ApoBf@NPs, R/E@NPs, or ApoBf-R/E@NPs over the indicated R/E-equivalent formulation concentrations. For unloaded ApoBf@NPs, carrier exposure was matched to the corresponding drug-loaded formulation. (D) Immunoblot analysis of the autophagy-associated proteins LC3B, SQSTM1/p62, and PSAT1 in LN229 cells following the indicated treatments. (E) Immunoblot analysis of ABCA1 abundance in LN229 cells. (F) Immunoblot analysis of the stemness-associated proteins NOTCH1, OCT4, KLF4, NANOG, and SOX2 in LN229 cells. (G–R) Flow-cytometric immunophenotyping following co-culture of pretreated GL261 cells with primary splenocyte and bone marrow-derived immune fractions isolated from syngeneic mice. Representative plots and corresponding quantification show CD80^+^CD86^+^ mature dendritic cells (G,H), iNOS^+^F4/80^+^ M1 macrophages (I,J), CD206^+^F4/80^+^ M2 macrophages (K,L), CD3^+^CD8a^+^ T cells (M,N), CD3^+^CD4^+^ T cells (O,P), and CD25^+^Foxp3^+^ regulatory T cells (Q,R). Numbers below immunoblot bands indicate densitometric values normalized to β-actin and expressed relative to the corresponding control.

**Fig. S10.** Dual-modal assessment of intracranial tumor-associated formulation distribution and quantitative analysis of in situ treatment responses. (A) Paired bioluminescence imaging of GL261-Luc tumors and DSPE-PEG-FITC-associated formulation fluorescence at 12 h after administration in the indicated treatment groups, together with corresponding ex vivo brain imaging. (B-F) Quantitative IHC scores for Ki-67 (B), Abca1 (C), Lc3b (D), Oct4 (E), and Sox2 (F) in orthotopic tumor sections corresponding to Fig. 8O,P. (G) Quantification of TUNEL-positive cells in orthotopic tumor sections corresponding to Fig. 8P.

**Fig. S11.** Systemic biochemical and histopathological safety assessment following treatment with ApoBf-R/E@NPs. (A-T) Serum biochemical analyses following the indicated treatments: amylase (AMY, A), albumin (ALB, B), alkaline phosphatase (ALP, C), alanine aminotransferase (ALT, D), aspartate aminotransferase (AST, E), total cholesterol (CHOL, F), creatine kinase-MB (CKMB, G), creatinine (Crea, H), C-reactive protein (CRP, I), direct bilirubin (DBil, J), glucose (GLU, K), high-density lipoprotein cholesterol (HDL, L), lactate dehydrogenase (LDH, M), low-density lipoprotein cholesterol (LDL, N), lipase (LIP, O), total bilirubin (TBil, P), triglycerides (TG, Q), total protein (TP, R), uric acid (UA, S), and urea (UREA, T). (U) Representative H&E staining of the brain, heart, and liver from the indicated treatment groups. (V) Representative H&E staining of the spleen, lung, and kidney. Scale bars, 100 μm.

**Fig. S12. Supplementary immunophenotyping and tissue-level analyses in the bilateral GL261 model.** **(A-F) Flow-cytometric immunophenotyping of the treated primary tumors, showing CD80^+^CD86^+^ mature DCs (A), iNOS^+^F4/80^+^ M1 macrophages (B), CD206^+^F4/80^+^ M2 macrophages (C), CD25^+^Foxp3^+^ Tregs (D), CD3^+^CD8a^+^ T cells (E), and CD3^+^CD4^+^ T cells (F), together with the corresponding quantification. (G-I) Flow-cytometric analysis of bone marrow-derived myeloid populations, including mature DCs (G), M1 macrophages (H), and M2 macrophages (I). (J-L) Flow-cytometric analysis of splenic lymphoid populations, including Tregs (J), CD8^+^ T cells (K), and CD4^+^ T cells (L).** (M-P) **Quantification of immunofluorescence-detected T-cell infiltration corresponding to Fig. 9P,Q: CD8^+^ (M) and CD4^+^ (N) T cells in distal tumors, and CD4^+^ (O) and CD8^+^ (P) T cells in primary tumors.** **(Q) Representative H&E staining of treated primary tumors. (R) Representative Ki-67 IHC staining of treated primary tumors. Scale bars, 100 μm. (S-U) Quantitative IHC scoring of Ki-67 (S), Bax (T), and Bcl-2 (U) in untreated distal tumors corresponding to Fig. 9R.** (V) Longitudinal body weight monitoring throughout the therapeutic window **in mice bearing bilateral tumors**.

**Fig. S13.** Supplementary assessment of postsurgical tumor control, immune remodeling, and systemic tolerance in the GL261-Luc recurrence model. (A) Representative gross images of the postoperative tumor beds following subtotal resection in the indicated groups. (B) Kaplan-Meier survival analysis of mice undergoing subtotal resection followed by the indicated postoperative treatments. (C) Representative H&E and Ki-67 staining and immunofluorescence imaging of CD8^+^ and CD4^+^ T cells in recurrent tumor tissues. Scale bar, 100 μm. (D-F) Quantification of Ki-67 IHC scores (D) and intratumoral CD8^+^ (E) and CD4^+^ (F) T-cell densities corresponding to panel C. (G) Longitudinal body-weight monitoring during postoperative treatment. (H-M) Flow-cytometric immunophenotyping of recurrent tumors, including CD80^+^CD86^+^ mature DCs (H), iNOS^+^F4/80^+^ M1 macrophages (I), CD206^+^F4/80^+^ M2 macrophages (J), CD25^+^Foxp3^+^ Tregs (K), CD3^+^CD8a^+^ T cells (L), and CD3^+^CD4^+^ T cells (M), together with the corresponding quantification. (N-P) Flow-cytometric analysis of bone marrow-derived myeloid populations, including mature DCs (N), M1 macrophages (O), and M2 macrophages (P). (Q-S) Flow-cytometric analysis of splenic lymphoid populations, including Tregs (Q), CD8^+^ T cells (R), and CD4^+^ T cells (S).

**Supplementary Table S1. Clinical and neuropathological characteristics of patient-derived GBM specimens.**

| **Study ID** | **Age** | **Sex** | **Tumor site** | **Pathological diagnosis** | **WHO grade** | **IDH1 R132H** | **ATRX** | **H3K27M** | **MGMT** | **Ki-67** | **p53** | **Study application** |
| --- | --- | --- | --- | --- | --- | --- | --- | --- | --- | --- | --- | --- |
| GBM106 | 50 | Female | Left corpus callosum | GBM | 4 | Negative | Retained | Negative | <1% positive | 70% | 80% positive | Paired TC/PBZ LDLR analysis; patient-derived GBM viability and CI analysis |
| GBM132 | 51 | Female | Right temporal lobe | GBM | 4 | Negative | Retained | Negative | NR | 40% (hot spot) | Wild-type pattern | Matched GBM/GSC/PBZ LDLR analysis; mechanistic and treatment-response studies |
| GBM152 | 68 | Male | Left temporal lobe | GBM | 4 | Negative | Retained | Negative | NR | 25% | 30% positive | Paired TC/PBZ LDLR analysis |
| GBM114 | 69 | Female | Right temporal lobe | GBM | 4 | Negative | Retained | Negative | NR | 40% (hot spot) | Wild-type pattern | Matched GBM/GSC/PBZ LDLR analysis |
| GBM178 | 75 | Female | Right temporo-occipital lobe | GBM | 4 | Negative | Retained | Negative | 15% positive | 20% | 25% positive | Paired TC/PBZ LDLR analysis; source tumor for GSC178 |

Abbreviations: CI, combination index; GBM, glioblastoma; GSC, glioma stem-like cell; IHC, immunohistochemistry; NR, not reported; PBZ, peritumoral brain zone; TC, tumor core.

Pathological and IHC features are transcribed from the corresponding institutional neuropathology reports. ATRX “retained” denotes positive nuclear staining as reported.

MGMT values denote MGMT protein IHC positivity reported in the pathology record and do not represent MGMT promoter methylation status.

Human specimens were obtained after written informed consent under approval no. 19K127-002 from the Ethics Committee of the First Hospital of Jilin University.

**Supplementary Table S2. Small interfering RNA (siRNA) sequences used for gene silencing in this study.**

| **siRNA** | | | | |
| --- | --- | --- | --- | --- |
| Target | Species |  | Sense (5'-3') | Antisense (5'-3') |
| ABCA1 | Human | #1 | GGUCAAACUUGAAGCUUCAAGAUUU | AAAUCUUGAAGCUUCAAGUUUGACC |
|  |  | #2 | CAGUACACAUUUGUCAGCAAUGAUG | CAUCAUUGCUGACAAAUGUGUACUG |
| Abca1 | Mouse | #1 | AACCACUUUGAUACUGAAUUA | UAAUUCAGUAUCAAAGUGGUU |
|  |  | #2 | UAGUCCUCUUUCUCGAAUUAU | AUAAUUCGAGAAAGAGGACUA |
| LDLR | Human | #1 | GGGCGACAGAUGCGAAAGAAA | UUUCUUUCGCAUCUGUCGCCC |
|  |  | #2 | ACAGAGGAUGAGGUCCACAUU | AAUGUGGACCUCAUCCUCUGU |
| Ldlr | Mouse | #1 | GCAACAUCUACUGGACAGAUU | AAUCUGUCCAGUAGAUGUUGC |
|  |  | #2 | GAUGUCAUAAACGAAGCCAUU | AAUGGCUUCGUUUAUGACAUC |
